# Allocation strategy of medical supplies during a public health emergency: a tripartite evolutionary game perspective

**DOI:** 10.1038/s41598-023-36000-y

**Published:** 2023-06-13

**Authors:** Youwei Yuan, Lanying Du, Lanjun Luo, Lei Cui

**Affiliations:** 1 School of Management, Huazhong University of Science and Technology, Wuhan, 430074 China; 2 School of Management and Economics, West Yunnan University, Lincang, 677000 China; 3 School of Management, North Sichuan Medical College, Nanchong, 637100 China; 4 School of Artificial Intelligence and Automation, Huazhong University of Science and Technology, Wuhan, 430074 China

**Keywords:** Natural hazards, Health care

## Abstract

Ensuring the rational and orderly circulation of medical supplies during a public health emergency is crucial to quickly containing the further spread of the epidemic and restoring the order of rescue and treatment. However, due to the shortage of medical supplies, there are challenges to rationalizing the allocation of critical medical supplies among multiple parties with conflicting interests. In this paper, a tripartite evolutionary game model is constructed to study the allocation of medical supplies in the rescue environment of public health emergencies under conditions of incomplete information. The game’s players include Government-owned Nonprofit Organizations (GNPOs), hospitals, and the government. By analyzing the equilibrium of the tripartite evolutionary game, this paper makes an in-depth study on the optimal allocation strategy of medical supplies. The findings indicate that: (1) the hospital should reasonably increase its willingness to accept the allocation plan of medical supplies, which can help medical supplies allocate more scientifically. (2) The government should design a reasonable reward and punishment mechanism to ensure the rational and orderly circulation of medical supplies, which can reduce the interference of GNPOs and hospitals in the allocation process of medical supplies. (3) Higher authorities should strengthen the supervision of the government and the accountability for loose supervision. The findings of this research can guide the government in promoting better circulation of medical supplies during public health emergencies by formulating more reasonable allocation schemes of emergency medical supplies, as well as incentives and penalties. At the same time, for GNPOs with limited emergency medical supplies, the equal allocation of emergency supplies is not the optimal solution to improve the efficiency of emergency relief, and it is simpler to achieve the goal of maximizing social benefits by allocating limited emergency resources to the demand points that match the degree of urgency. For example, in Corona Virus Disease 2019, emergency medical supplies should be prioritized for allocation to government-designated fever hospitals that are have a greater need for medical supplies and greater treatment capacity.

## Introduction

The world has suffered tremendous losses in the nearly three years that Corona Virus Disease 2019 (COVID-19) has been spreading. Globally, there have been more than 600 million confirmed cases of COVID-19 until September 6, 2022, including approximately 6.5 million deaths, reported to the World Health Organization (WHO)^1^. On January 30, 2020, the WHO Director-General declared COVID-19 a Public Health Emergency of International Concern (PHEIC)^2^. PHEIC is an event that causes serious harm to the lives and health of the public; an infectious disease epidemic that has a significant impact; food and occupational poisoning; unexplained mass diseases; and other events that seriously affect the safety of human life and health. Public health emergencies, such as COVID-19, have a huge impact on the health of human beings and the development of the world economy. Therefore, we must take emergency response measures to deal with it.

The standard emergency supplies consist of the following necessary players: the Government-owned Nonprofit Organizations (GNPOs), the hospital, the community, and the government. The GNPOs such as the Red Cross Society of China, act as buffer zones for emergency supplies such as medical supplies and are responsible for mobilizing the collection and allocation of related supplies. The hospitals, which are important parts of the response to public health emergencies, have a competitive relationship with GNPOs regarding the allocation of medical supplies due to their surging demand for medical supplies^3,4^. Hospitals demand medical supplies that can quickly and maximally meet their needs in the fight against the epidemic, but this conflicts with the GNPO’s limited dispatching capacity. The participation of the government in supervising the process of medical supplies allocation can ensure openness, fairness and impartiality in the process of supplies allocation, as well as effectively avoid the waste of medical supplies and fully utilize the benefits of medical supplies.

However, the GNPO’s task of allocating resources has not been effectively carried out. Due to the extreme urgency and severity of public health emergencies, the entire community will be in urgent demand for medical supplies, which will lead to problems in the timeliness and fairness of medical supplies allocation by GNPOs. Accordingly, this research aims to investigate the optimal allocation of medical supplies in a context that includes GNPOs, hospitals, and the government. In particular, we are interested in the following questions that are not fully explored: (1) How will GNPOs and hospitals determine the allocation of medical supplies? (2). How should the government, as the organizer and commander of the emergency management system in China, intervene to make the allocation plan acceptable to GNPOs, hospitals, and the government? The answers to these questions are of great importance for the rapid control of the epidemic and the safeguarding of people’s lives and health.

For the subject of supply allocation in emergencies, the current research can be divided into two categories: decision optimization for the allocation planning of emergency supplies and the construction of classical game models in the process of emergency supply allocation. The research on decision optimization of emergency supply allocation planning focuses primarily on the optimization of multi-period allocation of emergency resources for collaborative rescue, multi-objective optimization of multi-period dynamic emergency resource scheduling, and a hybrid fuzzy clustering optimization method for improving emergency logistics distribution^5–8^. But the method of using optimization theory to determine the allocation plan ignores the strategic interactions among the players in the allocation process^9–11^. The research on the construction of classical game models in the process of emergency supply allocation mainly focuses on the game model combined with the resource allocation algorithm and the optimal resource allocation plan obtained through a finite sequence game, ignoring the existence of multiple stages in the game process^12–17^. Therefore, it is necessary to study the evolutionary process of the game to provide support for the allocation of emergency supplies.

Because of the sudden and rapid nature of public health emergencies, emergency supply decision makers lack the necessary information when responding and must take measures while continuing to search for information. The measures taken at this time are necessarily limited. At the same time, they also have to make timely adjustments in the decision-making process according to the feedback provided by relevant stakeholders on the decision. As a result, the process of allocating emergency supplies during public health emergencies can be viewed as a game involving cooperation, conflict, and interaction. Game theory is the study of the problem of analyzing the decision process and equilibrium of parties in a conflict or cooperation with mutual influence of interests when there is interaction between two or more decision subjects and the decision strategy of any party cannot be completely independent of the strategies of the other parties. John Von Neumann and others led the development of game theory, and they established an important link between game theory and economics^18^. The game theory before evolutionary games is often regarded as “classical game theory” in economics. An important milestone in the development of classical game theory is the strategic equilibrium of non-cooperative games introduced by John Forbes Nash^19^. Selten and Harsanyi et al.^20,21^ extended the idea of Nash equilibrium to dynamic and incomplete information games. Classical game theory requires that all players be completely rational. However, due to the contingency and sudden change of public health emergencies, the incompleteness of information, and the difference in interest motivation among game players, the decision-making departments and related stakeholder groups show the characteristics of “bounded rationality”. Therefore, classical game theory is inapplicable to the process of allocating emergency supplies during public health emergencies. At the same time, the emergency nature of the situation also makes the game players incapable of getting the optimal game strategy through repeated inferences but need to seek it through continuous imitation learning in multiple games^22^.

Evolutionary game theory, built on the assumptions of bounded rationality, can transform the behavioral model of game players into an asymptotic evolutionary process of adaptive adjustment^23^. It first originated in the game analysis of conflict and cooperative behavior of plant and animal populations by genetic ecologists until Smith et al. creatively proposed the evolutionary stabilization strategy (ESS), which marked the formal birth of evolutionary game theory^24^. Another breakthrough concept of evolutionary game theory, replicator dynamic, was first introduced by Tylor et al. in their study of ecological evolution^25^, and then the replicator dynamic equation was extended from the symmetric case to the asymmetric case^26^. ESS and replicator dynamic constitute the core concepts of evolutionary game theory, and they represent the steady state and dynamic convergence processes of evolutionary games, respectively. Weibull summarized evolutionary game theory more systematically in his book^27^. At the same time, many scholars have applied evolutionary game theory to different fields, especially the economic field. For example, Friedman used the evolutionary game approach to analyze the evolution of Japanese and American firm organization patterns in the presence and absence of trade^28^. In evolutionary game theory, the optimal strategies of game players are obtained through imitative learning rather than being derived through inductive projection in a completely rational manner. This makes up for the shortcomings of classical game theory^29^, and it is also more consistent with the evolutionary characteristics of the behavioral strategies of GNPOs, hospitals, and government supervisors in the allocation process of medical supplies during a public health emergency.

In the study of emergency resource allocation, evolutionary game theory has a wide range of applications. Zhang et al.^30^ constructed an evolutionary game model of government, business, and society in a natural disaster situation and demonstrated that cooperation between government and business is beneficial in supplementing the massive demand for emergency supplies in disaster areas during sudden disasters. Ma et al.^31^ built a tripartite evolutionary game model of government, social organizations, and the public to study the impact of the blockchain platform on emergency supplies allocation, and the results showed that increasing government penalties improved the probability of active allocation by social organizations and strict supervision by government. The cooperation of relevant stakeholders in the supply chain is important for the successful achievement of resource supply goals^32^. Hu et al.^33^ investigated the different behavioral strategies of game players for the allocation process of emergency resources at different stages of the crisis by constructing a tripartite evolutionary game model of emergency collaboration among government, retailers, and suppliers in a crisis situation.

Based on the above analysis, this paper intends to study the game problem of GNPOs, hospitals, and the government in the allocation process of medical supplies during a public health emergency. To address this issue, this paper first develops a tripartite evolutionary game model of GNPO-hospital-government in the context of public health emergencies to investigate the factors influencing the allocation of medical supply by GNPO. Secondly, it conducts simulation experiments using real cases and conducts parametric sensitivity analysis on relevant influencing factors. And the impact of government incentives and penalties on the project’s performance is discussed. This study provides some reference for the rational circulation of medical supplies in the context of public health emergencies.

The following are the main contributions of this paper:

1. Under the assumption of "bounded rationality", this paper applies the evolutionary game approach to the circulation of medical supplies in public health emergencies and constructs a tripartite game model of GNPO-hospital-government. This study provides theoretical support for the allocation of medical supplies during public health emergencies.
2. This paper has studied the important factors that influence the allocation process of emergency medical supplies during public health emergencies.
3. In the part of numerical simulation, this paper adopts relevant parameters based on realistic situations, such as the government’s penalty for GNPO, the government’s penalty for the fighting of hospitals, and the government’s reward for hospitals’ acceptance of an unscientific medical supply allocation plan, etc. The results of the research are obtained by using data from real-life situations as cases, which can provide more feasible practical guidance for similar problems in the future.
4. Although the GNPO is a type of nonprofit organization unique to China, the results of the research can provide some reference for organizations that play the same role as GNPOs in achieving a more rational circulation of medical supplies during public health emergencies around the world.

## Literature review

Since the outbreak of COVID-19, the allocation of emergency resources in public health emergencies has received increasing attention from scholars, the public, and government departments. The literature review in this section is divided into two main parts: the allocation of emergency resource and evolutionary game theory and its application.

### The allocation of emergency resource

In public health emergencies, the reasonable allocation of emergency resources is very important for reducing the losses of the affected people and ensuring the smooth implementation of rescue activities. Due to the inherent real-time nature, uncertainty, and scarcity of the process of emergency resource allocation, in order to maximize the emergency resources to meet the needs of the affected people, it is necessary to reasonably coordinate the relevant stakeholders involved in the rescue process^34^.

It is the importance of the problem of emergency resource allocation in public health emergencies that has led to a great deal of research by scholars. The relevant research is divided into two main categories: optimization problems and game problems. Some scholars regard it as an optimization problem for the allocation of emergency resources under uncertain demand. Wang proposed an optimization model for multi-period allocation of emergency resources based on regional self-help and cross-regional collaborative rescue^9^. Zhou et al.^10^ designed a multi-objective optimization model for the multi-period dynamic Emergency Resource Scheduling (ERS) problem in disaster resource allocation. Ji et al.^35^ developed a bi-objective optimization model for maximizing the time-varying fill rate of emergency relief demand and minimizing the time-varying window of emergency relief allocation.

Other scholars have explored the optimal allocation strategy for emergency resources by constructing a game model. Gupta et al.^12^ constructed a game-theoretic framework using a self-developed resource allocation algorithm. Hu^36^ studied different resource allocations for rewarding endowments in collective risk social dilemmas through the analysis of public goods games and discussed their effects on the evolution of public cooperation and the accumulation of common resources in structured populations.

In summary, for the allocation of emergency resources, scholars mainly studied the problem by constructing optimization models and game models. The first one was to construct an optimization model to optimize the allocation of emergency resources under the situation of uncertain demand. The second kind of research mainly explored the optimal strategies of the game players in the emergency resource allocation system by constructing a game model. However, the existing studies mainly discussed the game from a static perspective or treat the stakeholders as independent individuals, without considering the interactive behavior of the allocation process and the bounded rationality of the stakeholders involved in this study. Therefore, this paper adopts the evolutionary game approach under the assumption of "bounded rationality" to explore the optimal strategies of the GNPO, hospital, and government in the process of allocating medical supplies during public health emergencies.

### Evolutionary game theory and its application

Evolutionary game theory is based on the assumption of "bounded rationality" and was first used to analyze the competitive behavior of different species or populations in an ecosystem. And through decades of development and improvement, the evolutionary game approach has been widely used in many disciplines. Han et al. constructed an evolutionary game model to explore the responses of firms under the interventions of low-carbon policy^37^. In addition, the evolutionary game approach has also been widely adopted in the study of complex interactions among players. To study the evolutionary pattern of construction workers’ unsafe behaviors during construction, Huang et al.^38^ constructed a two-sided evolutionary game model of construction workers and managers. The results showed that both higher bonuses and higher fines could reduce workers’ willingness to choose unsafe behaviors.To study the problem of organizational coordination in sustainable humanitarian supply chains, Zhang et al.^39^ constructed an evolutionary game model considering traditional mechanism and trust mechanism, and simulated the model using hypothetical data. In order to study the relationship between local governments and social organizations in the event of a natural disaster, Liu et al.^40^ constructed an emergency incentive game model and an emergency linkage game model for natural disaster emergency response using the evolutionary game approach. Yuan et al.^22^ constructed an evolutionary game model of the GNPO and hospital in the context of a public health emergency, and the stable strategic behavior of the game system was analyzed.

The analysis of evolutionary games with multiple players is more helpful to delineate complex problems, and the tripartite evolutionary game is an effective method to study the dynamic changes of the multiple players’ strategies with bounded rationality in long-term repeated games. Zhu et al.^41^ constructed a stochastic evolutionary game model of local government-commercial bank-lending enterprises to study the dilemma problem in the process of green credit development. Zhou investigated the tripartite game problem in an environmental control context. The paper constructed a tripartite evolutionary game model of wastewater treatment enterprises-government-public, and the research results showed that the reward and punishment mechanism of the government plays an important role in environmental pollution control^42^. Fei et al.^43^ constructed a tripartite evolutionary game model of medical administrative organization-medical institutions-insured individuals to study the fraud problem in China’s health insurance system. To discuss the cooperation of stakeholders in public health emergencies, Xu et al.^44^ constructed a tripartite evolutionary game model of enterprise-local government-public. The results showed that reasonable subsidies and penalties can help tripartite cooperation to battle the epidemic. By constructing a tripartite evolutionary game model of the government, community, and residents under public health emergencies, Fan et al. demonstrated that dynamic reward and punishment mechanisms can play an effective role in suppressing the fluctuation problem in the game process under static situations^45^. Ouyang et al.^46^ constructed a tripartite evolutionary game model of government agencies, the Internet media, and the public to study the collaborative prevention and control problem during public health emergencies.

By combing through the relevant literature, there are several differences. Firstly, the existing studies had mainly focused on the application of the game approach to commercial behavior in order to maximize commercial interests, and they focused less on the circulation of public emergency medical supplies. Secondly, few studies had discussed the competition in the allocation of medical supplies in the specific context of public health emergencies, and there was a lack of research on the strategic choices, influencing factors, and stability of strategies of GNPOs, hospitals, and the government in the allocation process of emergency supplies. As a result, in order to analyze the complex behavioral relationship among GNPO, hospital, and the government during public health emergencies, this paper constructs a tripartite evolutionary game model of GNPO-hospital-government to investigate how to make medical supplies flow more scientifically during public health emergencies and maximize the overall benefits to society by analyzing the factors influencing the strategy choices of the three players, with the goal of providing assistance in the treatment of public health emergencies.

## Model assumptions and construction

**Figure 1:**
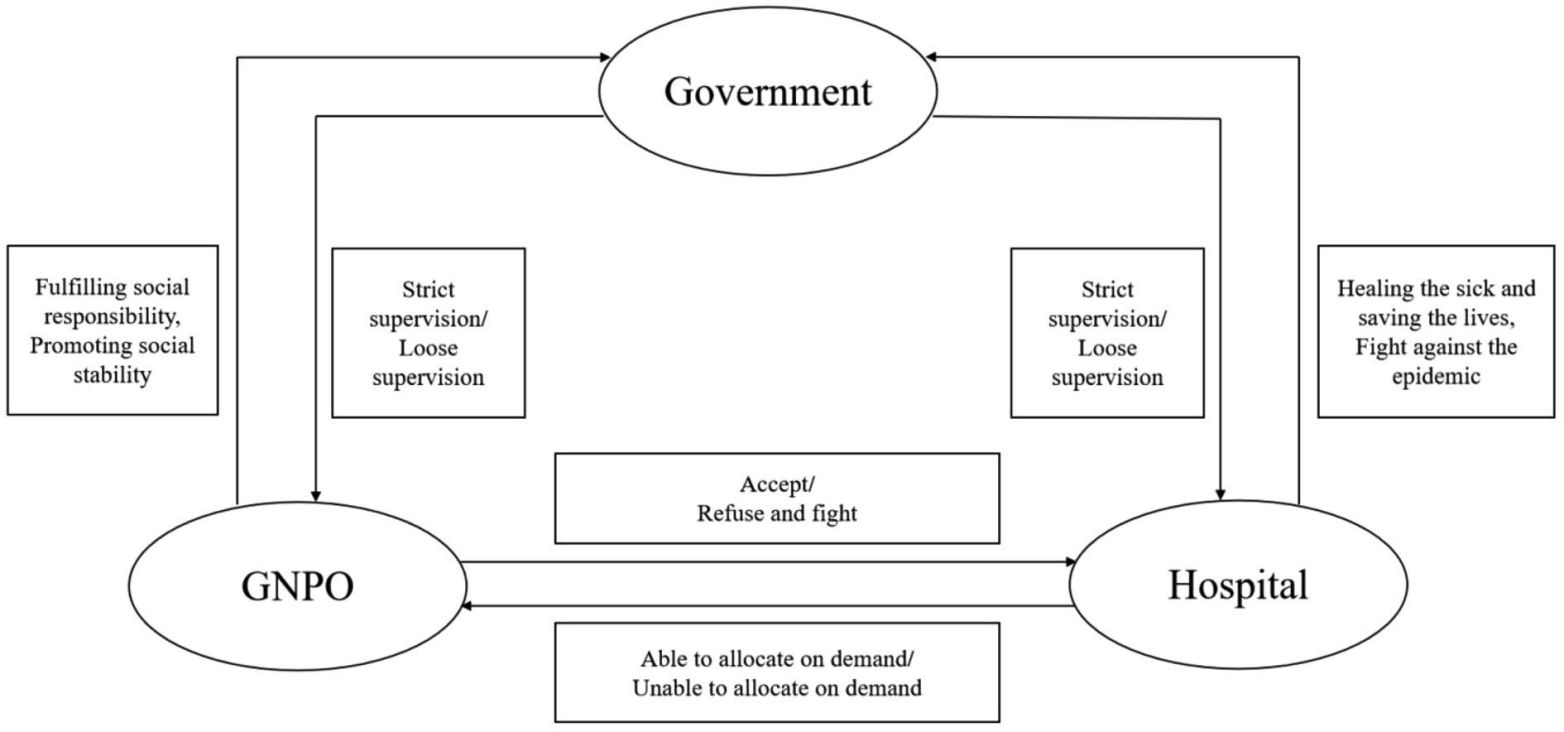
Logic relationship diagram of the tripartite evolutionary game model.

The logical relationship among the tripartite evolutionary game players of medical supplies circulation constructed in this paper is shown in Fig. 1.

### Model assumptions

We make the following assumptions based on the GNPO, hospital, and government problems with the circulation of medical supplies during public health emergencies.

#### Assumption 1

The players in the game, which include the GNPO, the hospital, and the government, are all finitely rational game subjects who cannot always make entirely rational decisions.

#### Assumption 2

The behaviors of the GNPO, hospital, and government are affected by each other. The GNPO will adjust its strategy of medical supplies allocation according to the government’s supervision strategy in order to optimize its own interests. Hospitals will also adjust their strategy choices based on the GNPO’s medical supplies allocation plan and the government’s supervision strategy. At the same time, the government also dynamically adjusts its supervision strategy according to the GNPO’s and the hospital’s strategy choices. Thus, they can dynamically optimize their strategy choices in the game process.

#### Assumption 3

When GNPOs do not allocate medical supplies scientifically, there is an opportunistic behavior of “free-riding”, which brings some additional benefits to GNPO such as direct over-allocation of medical supplies to individuals to enhance their social reputation^47^.

#### Assumption 4

The hospital will bear greater losses if the following two scenarios occur.

1. When the GNPO takes the strategy of “able allocate on demand” under strict government regulation, the hospital refuses the allocation plan and fights to gain more benefits.
2. When the GNPO takes the strategy of “unable allocate on demand”, the hospital accepts the allocation plan for the sake of optimizing the total benefit to society.

#### Assumption 5

GNPO’s strategy space is (able to allocate on demand, unable to allocate on demand). The strategy of “able to allocate on demand” means that the medical supplies allocated by GNPO can meet the emergency demand of the hospital as much as possible, considering the reported medical supplies demand of the hospital and the emergency level of the epidemic in the public health emergency. The strategy of “unable to allocate on demand” refers to the fact that although the hospitals report the demand for medical supplies during a public health emergency, the medical supplies allocation plan formulated by the GNPO cannot fully meet the emergency demand of the hospitals due to the severe shortage of medical supplies at this time and in order to meet the goal of optimizing the overall benefit to society in view of the urgency of the epidemic.

The hospital’s strategy space is (accept, refuse and fight). The strategy of “accept” means that the hospital chooses to accept whatever medical supplies allocation plan the GNPO has formulated. Even though there may be an allocation plan that does not fully meet the emergency demand of the hospital, the hospital still chooses to accept the plan in consideration of the goal of optimizing the overall social benefits and the stability of the epidemic control situation. The strategy of “refuse and fight” means that the hospital thinks that the GNPO’s allocation plan cannot meet its emergency demand, and the hospital that is dissatisfied with the allocation plan chooses to fight with the GNPO or expose the GNPO’s allocation plan to the public.

The government’s strategy space is (strict supervision, loose supervision), and the introduction of regulatory mechanisms can greatly facilitate cooperation among individuals in the system, which can maximize the benefits of the system^48^.The strategy of “strict supervision” means that the government strictly controls GNPO’s supplies allocation plan and hospitals’ behavior involving medical supplies, and will punish GNPO in the case of an unscientific medical supplies allocation plan. At the same time, if the hospitals are dissatisfied with GNPO’s supplies allocation plan and choose to fight with the GNPO or expose themselves to the public, the government will penalize the hospital, considering that such behavior is not conducive to the control of the epidemic and will seriously affect the stability of society during the period of epidemic control. When the government chooses the strategy of “loose supervision”, the government has no information about the strategic choices of GNPOs and hospitals, so the government does not reward or punish GNPOs and hospitals for their behavior at this time.

#### Assumption 6

Probability parameters for strategy selection by the game players. Assume that the probability that GNPO chooses the strategy of “able to allocate on demand” is x, where 0 ≤ x ≤ 1, and the probability that GNPO chooses the strategy of “unable to allocate on demand” is 1 − x. Similarly, the probability that the hospital chooses the strategy of “accept” is y, where 0 ≤ y ≤ 1, and the probability that it chooses the strategy of “refuse and fight” is 1 − y. The probability that the government chooses the strategy of “strict supervision” is z, where 0 ≤ z ≤ 1, and the probability that it chooses the strategy of “loose supervision” is 1 − z.

#### Assumption 7

Due to the different identities and priorities of GNPO, hospital and the government, in the emergency time of epidemic prevention and control, some hospitals may choose the strategy of “refuse and fight” in order to gain more benefits such as medical supplies after getting a reasonable allocation of medical supplies from GNPO. This will result in the GNPO’s social reputation being affected, and the resulting loss will be recorded as V_1_. At this point, if the government chooses to strictly supervise the flow of medical supplies in society, the hospital that chooses the strategy of "”refuse and fight” will be punished (denoted as P_1_)^49^. At the same time, the hospital which chooses the strategy of “refuse and fight” will receive additional supplies compensation from the GNPO (denoted as W_1_), considering the purpose of maintaining the stability of the epidemic prevention situation and the rapid conflict resolution by the GNPO. In the process of fighting, the hospitals need to pay for time and energy (denoted as H_2_), and GNPO needs to pay for coordination with the hospitals (denoted as H_1_). And α is the degree of the hospital’s fighting.

When the GNPO formulates an unscientific allocation plan for medical supplies, the hospital accepts the unscientific allocation plan considering the goal of optimizing the overall social benefit and the stability of the epidemic prevention and control situation. This results in some additional losses to the hospital (denoted as D). Meanwhile, the government, which chooses the strategy of “strict supervision” rewards the hospital (denoted as B) and penalizes the GNPO (denoted as K_2_). At the same time, the opportunistic behavior of formulating an unscientific medical supplies allocation plan for the hospital will result in some additional gain for the GNPO (denoted as A)^50^. However, since the hospital’s behavior of fighting at this point has a negative impact on social stability, the government that chooses the strategy of “strict supervision” also penalizes the GNPO (denoted as K_1_). The hospital’s strategy of “refuse and fight” will lead to the exposure of the GNPO’s unscientific behavior, which will cause the public to be dissatisfied with the GNPO and lead to greater losses such as a decrease in the GNPO’s credibility and a loss of public trust (denoted as V_2_, V_2_ > V_1_).In the meantime, the hospital that chooses the strategy of “refuse and fight” will receive additional supplies compensation from the GNPO (denoted as W_2_, W_2_ > W_1_). Although the hospital’s behavior of fighting will make society aware of the GNPO’s unscientific supply allocation plan, that will also have some negative impact on the epidemic prevention and control situation at this point. For the sake of maintaining the general situation of social stability, the government that chooses the strategy of "strict supervision" will also punish the hospital (denoted as P_2_, P_1_ > P_2_)^51^.

#### Assumption 8

In the emergency time of epidemic prevention and control, when the GNPO allocates medical supplies scientifically, the epidemic transmission chain can be cut off quickly because patients can be treated in time, and thus the epidemic will be controlled quickly. This will bring social benefits to the government (denoted as R_1_) as it strongly contributes to the subsequent economic development and social stability. Meanwhile, if the government chooses to strictly supervise the flow of medical supplies, this will greatly enhance the motivation and confidence of the public in fighting the epidemic. This will bring the government the benefit of increased social credibility (denoted as R_2_). When the GNPO’s allocation of medical supplies is unscientific, it is extremely detrimental to the control of epidemic and has a negative impact on social stability and economic development. In order to maintain social stability and promote economic recovery, the government needs to make more efforts to control the epidemic and pacify the public, the cost of which is H_3_. When the government strictly supervises the behavior of GNPOs and hospitals, the cost of which is H_4_. In addition, the government’s game strategy may have the “free-rider” mentality. If the GNPO allocates medical supplies scientifically and the hospital accepts GNPO’s plan, the government may choose the strategy of “loose supervision”. The cost that the government pays when it chooses the strategy of “loose supervision” is H_5_ (H_4_ > H_5_)^52^. At this moment, the government has no information about the strategy choices of GNPOs and hospitals, so it does not reward or punish the behavior of GNPOs and hospitals. However, when the government is loose with supervision, it leads to a lack of supervision, and hospitals may be dissatisfied with the medical supply allocation plan and fight. Since the hospital’s behavior of fighting has a serious negative impact on social stability, the government will be held accountable by higher authorities. We set the loss of the government department due to the accountability of the higher authorities as T (T > H_4_).

The relevant variables and their explanations are shown in Table 1 below, involving non-negative real numbers for all relevant variables. As game theory is usually based on simplifying assumptions and theoretical models to analyze the actual problem, the dependent relationship between variables is usually not considered.

**Table 1:** Variables and explanations.

Item	Variables	Explanations
For GNPO	K_1_	Punishment by the government for GNPO due to the hospital’s strategy of “refuse and fight” under strict government supervision
	K_2_	Punishment by the government for GNPO who chooses the strategy of “unable to allocate on demand” under strict government supervision
	V_1_	Losses resulting from the decline of the GNPO’s social reputation due to the hospital’s strategy of “refuse and fight” when the GNPO chooses the strategy of “able to allocate on demand”
	V_2_	Greater losses resulting from the decline of GNPO’s credibility and the loss of public trust due to the hospital’s strategy of “refuse and fight” when the GNPO chooses the strategy of “unable to allocate on demand” (V_2_ > V_1_)
	A	Additional gain for GNPO due to the opportunistic behavior of choosing the strategy of “unable to allocate on demand”
	H_1_	Coordination costs for GNPOs because the hospital chooses the strategy of “refuse and fight”
For hospital	D	Additional losses incurred by hospitals due to the GNPO’s strategy of “unable to allocate on demand” in the early stage of the epidemic
	E	Additional losses incurred by hospitals in the early stage of the epidemic due to the severe shortage of medical supplies in society and the difficulty in ensuring the supply of medical supplies (D > E)
	$\mathrm{\alpha }$	Degree of fighting by hospital
	W_1_	Additional supplies gain from GNPO due to the hospital’s strategy of “refuse and fight” when the GNPO chooses the strategy of “able to allocate on demand” under strict government supervision
	W_2_	Additional supplies gain from GNPO due to the hospital’s strategy of “refuse and fight” when the GNPO chooses the strategy of “unable to allocate on demand” under strict government supervision (W_1_ < W_2_)
	B	Incentive from the government because the hospital accepts the GNPO’s allocation plan when the GNPO chooses the strategy of “unable to allocate on demand” under strict government supervision
	P_1_	Punishment from the government due to the hospital’s strategy of “refuse and fight” when the GNPO chooses the strategy of “able to allocate on demand” under strict government supervision
	P_2_	Punishment from the government due to the hospital’s strategy of “refuse and fight” when the GNPO chooses the strategy of “unable to allocate on demand” under strict government supervision (P_1_ > P_2_)
	H_2_	Coordination costs for the hospital due to choosing the strategy of “refuse and fight”
For Government	R_1_	GNPO’s strategy of “able to allocate on demand” is conducive to the rapid control of the epidemic, promoting subsequent economic development and stability, and bringing social benefits to the government
	H_3_	GNPO’s strategy of “unable to allocate on demand” hinders the rapid control of the epidemic. In order to maintain social stability and promote early economic recovery, the government needs to make more efforts to control the epidemic and pacify the public, the cost of which is H_3_
	H_4_	The cost of strict government supervision is H_4_
	H_5_	The cost of loose government supervision is H_5_ (H_4_ > H_5_)
	R_2_	In the emergency situation, strict government supervision will greatly enhance the public’s motivation to fight the epidemic and their confidence to overcome it, thus bringing the government the benefit of improving social credibility
	T	When the government chooses the strategy of “loose supervision”, the government will be held accountable by higher authorities due to the serious negative impact of the hospital’s fighting on social stability, and the loss due to the accountability of higher authorities will be T (T > H_4_)
	B	Incentive by the government for hospital because the hospital accepts the GNPO’s allocation plan when the GNPO chooses the strategy of “unable to allocate on demand” under strict government supervision
	P_1_	Punishment by the government for hospital due to the hospital’s strategy of “refuse and fight” when the GNPO chooses the strategy of “able to allocate on demand” under strict government supervision
	P_2_	Punishment by the government for hospital due to the hospital’s strategy of “refuse and fight” when the GNPO chooses the strategy of “unable to allocate on demand” under strict government supervision (P_1_ > P_2_)
	K_1_	Punishment by the government for GNPO due to the hospital’s strategy of “refuse and fight” under strict government supervision
	K_2_	Punishment by the government for GNPO who chooses the strategy of “unable to allocate on demand” under strict government supervision

### Model construction

By setting the relevant variables and their explanations above, we can construct the tripartite evolutionary game payoff matrix of the GNPO, hospital, and government in the process of medical supplies allocation during a public health emergency, as shown in Table 2.

**Table 2:** The payoff matrix of tripartite evolutionary game.

		Hospital	Government	
			Strict supervision	Loose supervision
GNPO	Able to allocate on demand	Accept	0	0
			− E	− E
			R_1_ + R_2_ − H_4_	R_1_ − H_5_
		Refuse and fight	− K_1_ − $\mathrm{\alpha }$ V_1_− $\mathrm{\alpha H}$_1_	− $\mathrm{\alpha }$ V_1_ − $\mathrm{\alpha H}$_1_
			$\mathrm{\alpha }$ W_1_− E− $\mathrm{\alpha P}$_1_− $\mathrm{\alpha H}$_2_	$\mathrm{\alpha }$ W_1_− E− $\mathrm{\alpha H}$_2_
			R_1_ + R_2_ + K_1_ + $\mathrm{\alpha P}$_1_ − H_4_	R_1_ − H_5_ − T
	Unable to allocate on demand	Accept	A − K_2_	A
			B− D	− D
			R_2_ + K_2_ − B− H_3_ − H_4_	− H_3_ − H_5_
		Refuse and fight	A− $\mathrm{\alpha }$ V_2_ − $\mathrm{\alpha H}$_1_ − K_1_ − K_2_	A − $\mathrm{\alpha }$ V_2_ − $\mathrm{\alpha H}$_1_
			$\mathrm{\alpha }$ W_2_ − D − $\mathrm{\alpha P}$_2_ − $\mathrm{\alpha H}$_2_	$\mathrm{\alpha }$ W_2_ − D − $\mathrm{\alpha H}$_2_
			R_2_ + K_1_ + K_2_+$\mathrm{\alpha P}$_2_ − H_3_ − H_4_	− H_3_ − H_5_ − T

### Model analysis

From the evolutionary principle of evolutionary game theory, it is known that when the expected payoffs obtained by a particular strategy chosen by game players are higher than the average payoffs of mixed strategies, then this particular strategy will tend to evolve in the system and the number of game players adopting that strategy will increase^53^. We use replicated dynamic differential equations to describe the frequency of change of this particular strategy in the system^54,55^.

### Strategy stability analysis of GNPO

From the payoff matrix, the expected return of GNPO when choosing the strategy of “able to allocate on demand” is

1 $$\begin{aligned} E_{11} & = yz*0 + y\left( {1 - z} \right)*0 + \left( {1 - y} \right)z\left( { - K_{1} - \alpha V_{1} - \alpha H_{1} } \right) + \left( {1 - y} \right)\left( {1 - z} \right)\left( { - \alpha V_{1} - \alpha H_{1} } \right) \\ & = z\left( {y - 1} \right)\left( {K_{1} + \alpha H_{1} + \alpha V_{1} } \right) - \left( {\alpha H_{1} + \alpha V_{1} } \right)\left( {y - 1} \right)\left( {z - 1} \right) \\ \end{aligned}$$

The expected return of GNPO when choosing the strategy of “unable to allocate on demand” is

2 $$\begin{aligned} E_{12} & = yz\left( {A - K_{2} } \right) + y\left( {1 - z} \right)A + \left( {1 - y} \right)z\left( {A - \alpha V_{2} - \alpha H_{1} - K_{1} - K_{2} } \right) + \left( {1 - y} \right)\left( {1 - z} \right)\left( {A - \alpha V_{2} - \alpha H_{1} } \right) \\ & = z\left( {y - 1} \right)\left( {K_{1} - A + K_{2} + \alpha V_{2} + \alpha H_{1} } \right) - \left( {y - 1} \right)\left( {z - 1} \right)\left( {\alpha H_{1} - A + \alpha V_{2} } \right) + yz\left( {A - K_{2} } \right) - Ay\left( {z - 1} \right) \\ \end{aligned}$$

Thus, the average return of GNPO under the mixed strategy is

3 $$\begin{aligned} E_{1} = & xE_{11} + \left( {1 - x} \right)E_{12} = x\left( {z\left( {y - 1} \right)\left( {K_{1} + \alpha V_{1} + \alpha H_{1} } \right) - \left( {\alpha V_{1} + \alpha H_{1} } \right)\left( {y - 1} \right)\left( {z - 1} \right)} \right) \\ & + \left( {x - 1} \right)\left( {\left( {y - 1} \right)\left( {z - 1} \right)\left( {\alpha V_{2} + \alpha H_{1} - A} \right) - z\left( {y - 1} \right)\left( {K_{1} - A + K_{2} + \alpha V_{2} + \alpha H_{1} } \right) - yz\left( {A - K_{2} } \right) + Ay\left( {z - 1} \right)} \right) \\ \end{aligned}$$

The replicated dynamic equation for the GNPO can be expressed as

4 $$F\left( x \right) = \frac{dx}{{dt}} = x(E_{11} - E_{1} ) = x\left( {1 - x} \right)\left[ {y\left( {\alpha V_{1} - \alpha V_{2} } \right) + \alpha V_{2} - \alpha V_{1} + zK_{2} - A} \right]$$

The first order derivative of x and the set G(y) are respectively

5 $$\frac{dF\left( x \right)}{{dx}} = \left( {2x - 1} \right)\left[ {y\left( {\alpha V_{2} - \alpha V_{1} } \right) + \alpha V_{1} - \alpha V_{2} - zK_{2} + A} \right]$$

6 $$G\left( y \right) = y\left( {\alpha V_{2} - \alpha V_{1} } \right) + \alpha V_{1} - \alpha V_{2} - zK_{2} + A$$

According to the principle of stability of differential equations, if the probability of the GNPO choosing the strategy of “able to allocate on demand” is in a stable state, it must satisfy that $\mathrm{F}\left(\mathrm{x}\right)=0$ and $\frac{d\mathrm{F}\left(\mathrm{x}\right)}{dx}<0$. $\mathrm{\alpha }{V}_{2}>\mathrm{\alpha }{V}_{1}$, then $\frac{\partial \mathrm{G}\left(y\right)}{\partial y}>0$. $\mathrm{G}\left(y\right)$ is an increasing function with respect to y. Therefore, when $y=\frac{\mathrm{\alpha }{V}_{1}-\mathrm{\alpha }{V}_{2}-z{K}_{2}+A}{\mathrm{\alpha }{V}_{1}-\mathrm{\alpha }{V}_{2}}={y}^{*}$, $\mathrm{G}\left(y\right)=0$. At this time,$\frac{d\mathrm{F}\left(\mathrm{x}\right)}{dx}\equiv 0$, $\mathrm{F}\left(\mathrm{x}\right)\equiv 0$, then all x values are in an evolutionary stable state. (1) When ${y<y}^{*}$, $\mathrm{G}\left(y\right)<0$.At this time, when $\mathrm{x}=1$, $\frac{d\mathrm{F}\left(\mathrm{x}\right)}{dx}<0$.So $\mathrm{x}=1$ is the evolutionary stable strategy of GNPO. (2) When ${y>y}^{*}$,$\mathrm{G}\left(y\right)>0$.At this time when $\mathrm{x}=0$, $\frac{d\mathrm{F}\left(\mathrm{x}\right)}{dx}<0$.So $\mathrm{x}=0$ is the evolutionary stable strategy of GNPO. That is, when the hospital has a high probability of choosing the strategy of “refuse and fight”, GNPO will tend to choose the strategy of “able to allocate on demand”.

**Figure 2:**
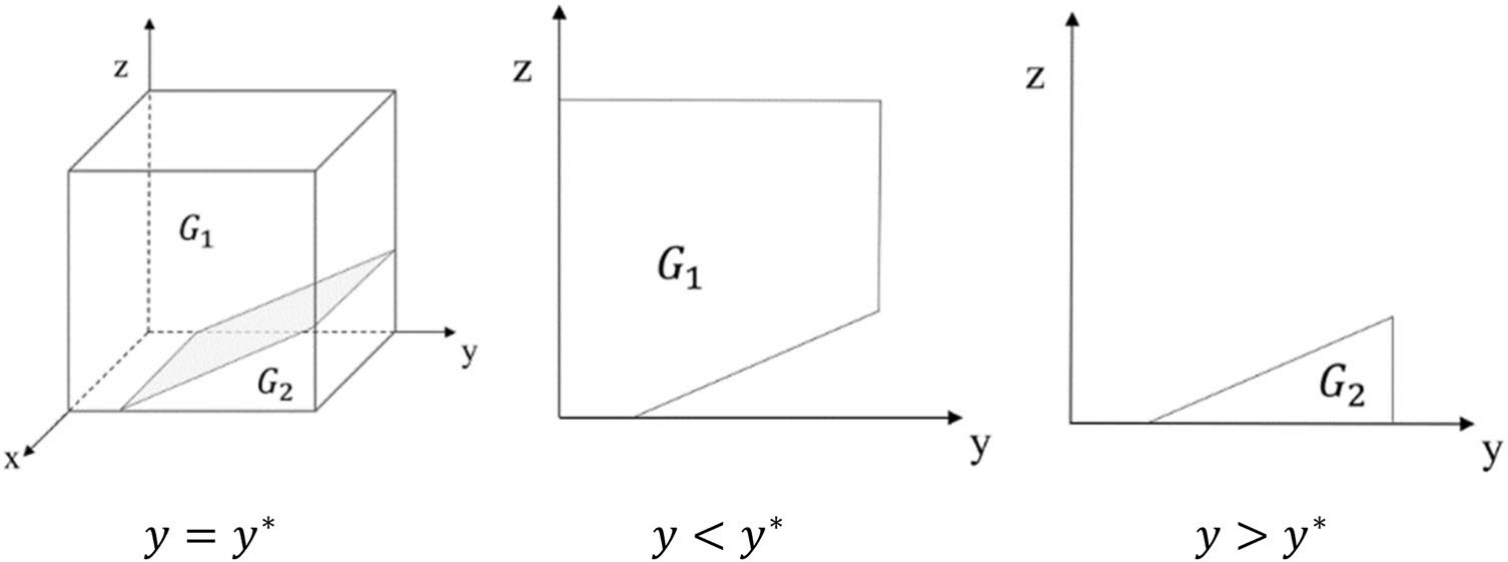
Phase diagram of GNPO strategy evolution.

The evolutionary phase diagram of GNPO’s strategy choice is shown in Fig. 2.

From Fig. 2, it is known that the tangent passes through the point (0, 1, $\frac{A}{{K}_{2}}$). In the tripartite evolutionary game, there exists a set surface consisting of undifferentiated evolutionary equilibrium points that cuts the set of strategy spaces into two parts, G_1_ and G_2_. The volume of part ${G}_{1}$ is the probability of GNPO choosing the strategy of “able to allocate on demand”, and we use ${V}_{{G}_{1}}$ to represent it. Meanwhile, the volume of part ${G}_{2}$ is the probability of GNPO choosing the strategy of “unable to allocate on demand”, and we use ${V}_{{G}_{2}}$ to represent it. From the formula, we can get:

7 $$V_{{G_{1} }} = \int_{{\frac{A}{{K_{2} }}}}^{1} {\int_{0}^{1} {\frac{{\alpha V_{1} - \alpha V_{2} - zK_{2} + A}}{{\alpha V_{1} - \alpha V_{2} }}dxdz = \frac{{\left( {A - K_{2} } \right)\left( {A - K_{2} + 2\alpha V_{1} - 2\alpha V_{2} } \right)}}{{2\alpha K_{2} \left( {V_{2} - V_{1} } \right)}}} }$$

8 $$V_{{G_{2} }} = 1 - V_{{G_{1} }} = 1 - \frac{{\left( {A - K_{2} } \right)\left( {A - K_{2} + 2\alpha V_{1} - 2\alpha V_{2} } \right)}}{{2\alpha K_{2} \left( {V_{2} - V_{1} } \right)}}$$

*Inference 1* The probability that the GNPO chooses the strategy of “able to allocate on demand” is positively correlated with respect to $\mathrm{\alpha }$ and K_2_, and negatively correlated with respect to A.

From Eq. (7), we can get:

$$\frac{{\partial V_{{G_{1} }} }}{\partial \alpha } > 0,\frac{{\partial V_{{G_{1} }} }}{{\partial K_{2} }} > 0,\frac{{\partial V_{{G_{1} }} }}{\partial A} < 0$$

The above equation shows that increasing $\mathrm{\alpha }$, K_2_, or decreasing A can increase the probability of GNPO choosing the strategy of “able to allocate on demand”.

In the emergency period of epidemic prevention and control, maximizing the utilization of medical supplies plays a very important role in quickly controlling the spread of the epidemic. Thus, it is important for the GNPO, as the allocator of medical supplies, to allocate medical supplies more scientifically to carry out the epidemic prevention and control work smoothly.

When the GNPO has an unscientific allocation plan due to chaotic work and other reasons, it may devote more time and energy to other epidemic prevention matters. At this time, the GNPO will gain certain extra benefits. By reducing the extra benefits gained by the GNPO through methods such as strict regulation of the GNPO’s work process, it can better promote the GNPO’s choice of scientific allocation of medical supplies at this moment. When the government strictly supervises the GNPO, increasing the amount of penalty for the unscientific allocation of medical supplies can encourage the GNPO to choose to allocate medical supplies scientifically. When a hospital receives an unscientific allocation plan, increasing the degree of fighting can also increase the probability that the GNPO chooses the strategy of “able to allocate on demand”, thus promoting the GNPO to allocate medical supplies more scientifically.

*Inference 2*: In the process of a tripartite evolutionary game, as the acceptance rate of hospitals for medical supplies allocation plan decreases or the rate of strict government supervision increases, the probability that the GNPO chooses the strategy of “able to allocate on demand” will increase.

From the strategy stability analysis of GNPO, it is known that when ${y<y}^{*}$, $\mathrm{G}\left(y\right)<0$.And x $=1$ is the evolutionary stability strategy of GNPO. When ${y>y}^{*}$, $\mathrm{G}\left(y\right)>0$. And x $=0$ is the evolutionary stability strategy of GNPO. In addition, because ${y}^{*}=\frac{\mathrm{\alpha }{V}_{1}-\mathrm{\alpha }{V}_{2}-z{K}_{2}+A}{\mathrm{\alpha }{V}_{1}-\mathrm{\alpha }{V}_{2}}$, the probability that GNPO’s stabilization strategy is x = 1 will be improved with the gradual decrease of y or gradual increase of z.

The above analysis shows that a reasonable reduction in the acceptance probability of medical supplies allocation plans by hospitals will help GNPOs make more efforts to make scientific allocation plans. The government can not only improve the probability of strict government supervision to rationalize the medical supplies allocation plan but also promote the overall benefit of society by playing the role of social forces to monitor the medical supplies allocation plan or appealing to the public to donate medical supplies. This can speed up the control of the epidemic and help win the battle against it as soon as possible.

### Strategy stability analysis of hospital

From the payoff matrix, the expected return of the hospital when choosing the strategy of “accept” is

9 $$\begin{aligned} E_{21} & = xz\left( { - E} \right) + x\left( {1 - z} \right)\left( { - E} \right) + \left( {1 - x} \right)z\left( {B - D} \right) + \left( {1 - x} \right)\left( {1 - z} \right)\left( { - D} \right) \\ & = x\left( {z - 1} \right)E - xzE - z\left( {x - 1} \right)\left( {B - D} \right) - D\left( {x - 1} \right)\left( {z - 1} \right) \\ \end{aligned}$$

The expected return of the hospital when choosing the strategy of “refuse and fight” is

10 $$\begin{aligned} E_{22} & = x\left( {z - 1} \right)\left( {E + \alpha H_{2} - \alpha W_{1} } \right) - xz\left( {E + \alpha H_{2} + \alpha P_{1} - \alpha W_{1} } \right) \\ & \quad + z\left( {x - 1} \right)\left( {D + \alpha H_{2} + \alpha P_{2} - \alpha W_{2} } \right) - \left( {x - 1} \right)\left( {z - 1} \right)\left( {D + \alpha H_{2} - \alpha W_{2} } \right) \\ \end{aligned}$$

Thus, the average return of the hospital under the mixed strategy is

11 $$\begin{aligned} E_{2} & = \left( {y - 1} \right)\left( {xz\left( {E + \alpha H_{2} + \alpha P_{1} - \alpha W_{1} } \right) - x\left( {z - 1} \right)\left( {E + \alpha H_{2} - \alpha W_{1} } \right) - z\left( {x - 1} \right)\left( {D + \alpha H_{2} + \alpha P_{2} - \alpha W_{2} } \right) + \left( {x - 1} \right)\left( {z - 1} \right)\left( {D + \alpha H_{2} + \alpha P_{2} - \alpha W_{2} } \right)} \right) \\ & \quad - y\left( {D\left( {x - 1} \right)\left( {z - 1} \right) + xzE + z\left( {B - D} \right)\left( {x - 1} \right) - x\left( {z - 1} \right)E} \right) \\ \end{aligned}$$

The replicated dynamic equation for the hospital can be expressed as

12 $$F\left( y \right) = \frac{dy}{{dt}} = y(E_{21} - E_{2} ) = y\left( {1 - y} \right)\left[ {z\left( {x*\alpha P_{1} - x*\alpha P_{2} - xB + B + \alpha P_{2} } \right) + x\left( {\alpha W_{2} - \alpha W_{1} } \right) + \alpha H_{2} - \alpha W_{2} } \right]$$

The first order derivative of y and the set $\mathrm{J}\left(x\right)$ are respectively

13 $$\frac{dF\left( y \right)}{{dy}} = \left( {2y - 1} \right)\left[ {z\left( {x*\alpha P_{2} - x*\alpha P_{1} + xB - B - \alpha P_{2} } \right) + x\left( {\alpha W_{1} - \alpha W_{2} } \right) + \alpha W_{2} - \alpha H_{2} } \right]$$

14 $$\left( {J\left( x \right) = z\left( {x*\alpha P_{2} - x*\alpha P_{1} + xB - B - \alpha P_{2} } \right) + x\left( {\alpha W_{1} - \alpha W_{2} } \right) + \alpha W_{2} - \alpha H_{2} } \right)$$

According to the principle of stability of differential equations, if the probability of hospital choosing the strategy of "accept" is in a stable state, it must satisfy that $\mathrm{F}\left(y\right)=0$ and $\frac{d\mathrm{F}\left(\mathrm{y}\right)}{dy}<0$.Because $\frac{\partial \mathrm{J}\left(x\right)}{\partial x}<0$, J$\left(x\right)$ is a decreasing function with respect to x. Therefore, there is when x $=\frac{z\left(\mathrm{B}+\mathrm{\alpha }{P}_{2}\right)-\mathrm{\alpha }{W}_{2}+\mathrm{\alpha }{H}_{2}}{\mathrm{z}*\mathrm{\alpha }{P}_{2}-\mathrm{z}*\mathrm{\alpha }{P}_{1}+\mathrm{zB}+\mathrm{\alpha }{W}_{1}-\mathrm{\alpha }{W}_{2}}={x}^{*}$,$\mathrm{J}\left(x\right)=0$. At this time,$\frac{d\mathrm{F}\left(\mathrm{y}\right)}{dy}\equiv 0$, $\mathrm{F}\left(\mathrm{y}\right)\equiv 0$, then all y values are in evolutionary stable state. (1) When ${x<x}^{*}$, $\mathrm{J}\left(x\right)>0$.At this time, when y $=0$, $\frac{d\mathrm{F}\left(\mathrm{y}\right)}{dy}<0$.So y $=0$ is the evolutionary stable strategy of hospital. (2) When ${x>x}^{*}$,$\mathrm{J}\left(x\right)<0$.At this time, when y $=1$, $\frac{d\mathrm{F}\left(\mathrm{y}\right)}{dy}<0$.So y $=1$ is the evolutionary stable strategy of hospital. That is, when the GNPO has a high probability of choosing the strategy of "able to allocate on demand", the hospital will tend to choose the strategy of "accept".

**Figure 3:**
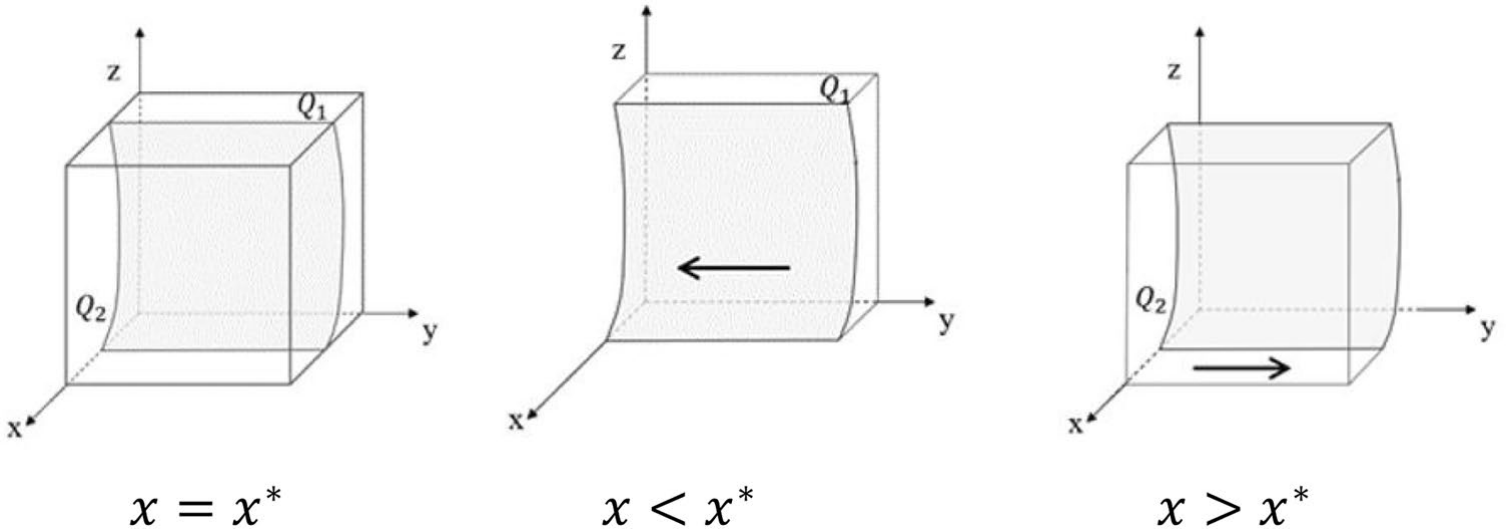
Phase diagram of hospital strategy evolution.

The evolutionary phase diagram of hospital’s strategy choice is shown in Fig. 3.

From Fig. 3, it is known that the volume of part ${Q}_{1}$ is the probability of the hospital choosing the strategy of “refuse and fight”, and we use ${V}_{{Q}_{1}}$ to represent it. Meanwhile, the volume of part ${Q}_{2}$ is the probability of the hospital choosing the strategy of “accept”, and we use ${V}_{{Q}_{2}}$ to represent it. From the formula, we can get:

15 $${V}_{{Q}_{1}}={\int }_{0}^{1}{\int }_{0}^{1}\frac{z\left(B+\alpha {P}_{2}\right)-\alpha {W}_{2}+\alpha {H}_{2}}{z*\alpha {P}_{2}-z*\alpha {P}_{1}+zB+\alpha {W}_{1}-\alpha {W}_{2}}dydz$$

16 $${V}_{{Q}_{2}}=1-{V}_{{Q}_{1}}$$

*Inference 3* The probability that the hospital chooses the strategy of "refuse and fight" is negatively correlated with respect to B, P_1_, and P_2_.

From Eq. (15), we can get:

$$\frac{\partial {V}_{{Q}_{1}}}{\partial B}<0,\frac{\partial {V}_{{Q}_{1}}}{\partial {P}_{1}}<0,\frac{\partial {V}_{{Q}_{1}}}{\partial {P}_{2}}<0$$

In the case of an epidemic outbreak, the hospitals, as receivers of medical supplies, choose to accept the allocation plans even if they are unscientific in order to achieve the maximum social benefits. The government that chooses the strict supervision strategy will give certain incentives to hospitals for their behavior when considering the overall situation. Increasing the number of incentives will reduce the probability that the hospitals will choose the strategy of “refuse and fight” when they receive unscientific allocation plans. At the same time, it is an emergency time for epidemic prevention and control, so the hospitals’ behavior of fighting will have a negative impact on social stability and epidemic prevention and control. For the sake of maintaining overall social stability, the government that chooses the strict supervision strategy will also punish the hospitals. Increasing the punishment amount will reduce the probability that the hospitals choose the strategy of “refuse and fight”.

*Inference 4* In the process of a tripartite evolutionary game, as the probability of the GNPO choosing the strategy of “able to allocate on demand” increases or the probability of the government choosing the strategy of “strict supervision” decreases, the probability of the hospital choosing the strategy of “accept” increases.

From the strategy stability analysis of the hospital, it is known that when ${x<x}^{*}$, $\mathrm{J}\left(x\right)>0$. And $\mathrm{y}=0$ is the evolutionary stability strategy of the hospital. When ${x>x}^{*}$, $\mathrm{J}\left(x\right)<0$. And $\mathrm{y}=1$ is the evolutionary stability strategy of the hospital. In addition, because ${x}^{*}=\frac{z\left(\mathrm{B}+\mathrm{\alpha }{P}_{2}\right)-\mathrm{\alpha }{W}_{2}+\mathrm{\alpha }{H}_{2}}{\mathrm{z}*\mathrm{\alpha }{P}_{2}-\mathrm{z}*\mathrm{\alpha }{P}_{1}+\mathrm{zB}+\mathrm{\alpha }{W}_{1}-\mathrm{\alpha }{W}_{2}}$, the probability that the hospital’s stabilization strategy is $\mathrm{y}=1$ will be improved with the gradual decrease of z or gradual increase of x.

The above analysis shows that the GNPO’s increase in the probability of choosing the strategy of “able to allocate on demand” during epidemic control emergencies is conducive to hospitals’ acceptance of the allocation plan, which will promote social stability and is important for the smooth implementation of epidemic control. In addition, the government’s tendency to choose the strategy of “loose supervision” may also lead hospitals to accept the GNPO’s medical supplies allocation plan.

### Strategy stability analysis of government

From the payoff matrix, the expected return of government when choosing the strategy of “strict supervision” is

17 $$\begin{aligned} E_{31} & = y\left( {x - 1} \right)\left( {B + H_{3} + H_{4} - R_{2} - K_{2} } \right) + \left( {x - 1} \right)\left( {y - 1} \right)\left( {K_{1} - H_{4} - H_{3} + R_{2} + \alpha P_{2} + K_{2} } \right) \\ & \quad - x\left( {y - 1} \right)\left( {K_{1} - H_{4} + R_{1} + R_{2} + \alpha P_{1} } \right) + xy\left( {R_{1} - H_{4} + R_{2} } \right) \\ \end{aligned}$$

The expected return of government when choosing the strategy of “loose supervision” is

18 $$E_{32} = x\left( {y - 1} \right)\left( {H_{5} - R_{1} + T} \right) - \left( {x - 1} \right)\left( {y - 1} \right)\left( {H_{3} + H_{5} + T} \right) + y\left( {x - 1} \right)\left( {H_{3} + H_{5} } \right) - xy\left( {H_{5} - R_{1} } \right)$$

Thus, the average return of government under the mixed strategy is

19 $$\begin{aligned} E_{3} = & \,zE_{31} + \left( {1 - z} \right)E_{32} = \left( {z - 1} \right)\left( {\left( {x - 1} \right)\left( {y - 1} \right)\left( {H_{3} + H_{5} + T} \right) - x\left( {y - 1} \right)\left( {H_{5} - R_{1} + T} \right) - y\left( {x - 1} \right)\left( {H_{3} + H_{5} } \right) + xy\left( {H_{5} - R_{1} } \right)} \right) \\ & + z\left( {y\left( {x - 1} \right)\left( {B + H_{3} + H_{4} - R_{2} - K_{2} } \right) + \left( {x - 1} \right)\left( {y - 1} \right)\left( {K_{1} - H_{4} - H_{3} + R_{2} + \alpha P_{2} + K_{2} } \right) - x\left( {y - 1} \right)\left( {K_{1} - H_{4} + R_{1} + R_{2} + \alpha P_{1} } \right) + xy\left( {R_{1} - H_{4} + R_{2} } \right)} \right) \\ \end{aligned}$$

The replicated dynamic equation for the government can be expressed as

20 $$\begin{aligned} F\left( z \right) & = \frac{dz}{{dt}} = z(E_{31} - E_{3} ) \\ & = z\left( {1 - z} \right)\left[ {R_{2} + K_{1} + K_{2} - H_{4} + H_{5} + T + \alpha P_{2} + x\left( {\alpha P_{1} - \alpha P_{2} - K_{2} } \right) + y\left( { - B - \alpha P_{2} - K_{1} - T} \right) + xy\left( {B - \alpha P_{1} + \alpha P_{2} } \right)} \right] \\ \end{aligned}$$

The first order derivative of z and the set $\mathrm{M}\left(y\right)$ are respectively

21 $$\frac{dF\left( z \right)}{{dz}} = \left( {2z - 1} \right)\left[ {H_{4} - R_{2} - K_{1} - K_{2} - H_{5} - T - \alpha P_{2} - x\left( {\alpha P_{1} - \alpha P_{2} - K_{2} } \right) - y\left( { - B - \alpha P_{2} - K_{1} - T} \right) - xy\left( {B + \alpha P_{2} - \alpha P_{1} } \right)} \right]$$

22 $$\begin{aligned} M\left( y \right) = & H_{4} - R_{2} - K_{1} - K_{2} - H_{5} - T - \alpha P_{2} - x\left( {\alpha P_{1} - \alpha P_{2} - K_{2} } \right) \\ & - y\left( { - B - \alpha P_{2} - K_{1} - T} \right) - xy\left( {B + \alpha P_{2} - \alpha P_{1} } \right) \\ \end{aligned}$$

According to the principle of stability of differential equations, if the probability of government choosing the strategy of "strict supervision" is in a stable state, it must satisfy that $$\mathrm{F}\left(z\right)=0$$ and $$\frac{d\mathrm{F}\left(\mathrm{z}\right)}{dz}<0$$.Because $$\frac{\partial \mathrm{M}\left(y\right)}{\partial y}>0$$, $$\mathrm{M}\left(y\right)$$ is an increasing function with respect to y. Therefore, there is when y $$=\frac{{H}_{4}-{R}_{2}-{K}_{1}-{K}_{2}-{H}_{5}-T-\mathrm{\alpha }{P}_{2}-x\left(\mathrm{\alpha }{P}_{1}-{\mathrm{\alpha }{P}_{2}-K}_{2}\right)}{x\left(B+\mathrm{\alpha }{P}_{2}-\mathrm{\alpha }{P}_{1}\right)-\mathrm{B}-\mathrm{\alpha }{P}_{2}-{K}_{1}-T}={y}^{**}$$,$$\mathrm{M}\left(y\right)=0$$. At this time,$$\frac{d\mathrm{F}\left(\mathrm{z}\right)}{dz}\equiv 0$$, $$\mathrm{F}\left(\mathrm{z}\right)\equiv 0$$, then all z values are in an evolutionary stable state. (1) When $${y<y}^{**}$$, $$\mathrm{M}\left(y\right)<0$$.At this time, when $$\mathrm{z}=1$$, $$\frac{d\mathrm{F}\left(\mathrm{z}\right)}{dz}<0$$.So $$\mathrm{z}=1$$ is the evolutionary stable strategy of government. (2) When $${y>y}^{**}$$,$$\mathrm{M}\left(y\right)<0$$.At this time, when $$\mathrm{z}=0$$, $$\frac{d\mathrm{F}\left(\mathrm{z}\right)}{dz}<0$$.So $$\mathrm{z}=0$$ is the evolutionary stable strategy of government. That is, when the hospital has a high probability of choosing the strategy of “accept”, government will tend to choose the strategy of “loose supervision”.

**Figure 4:**
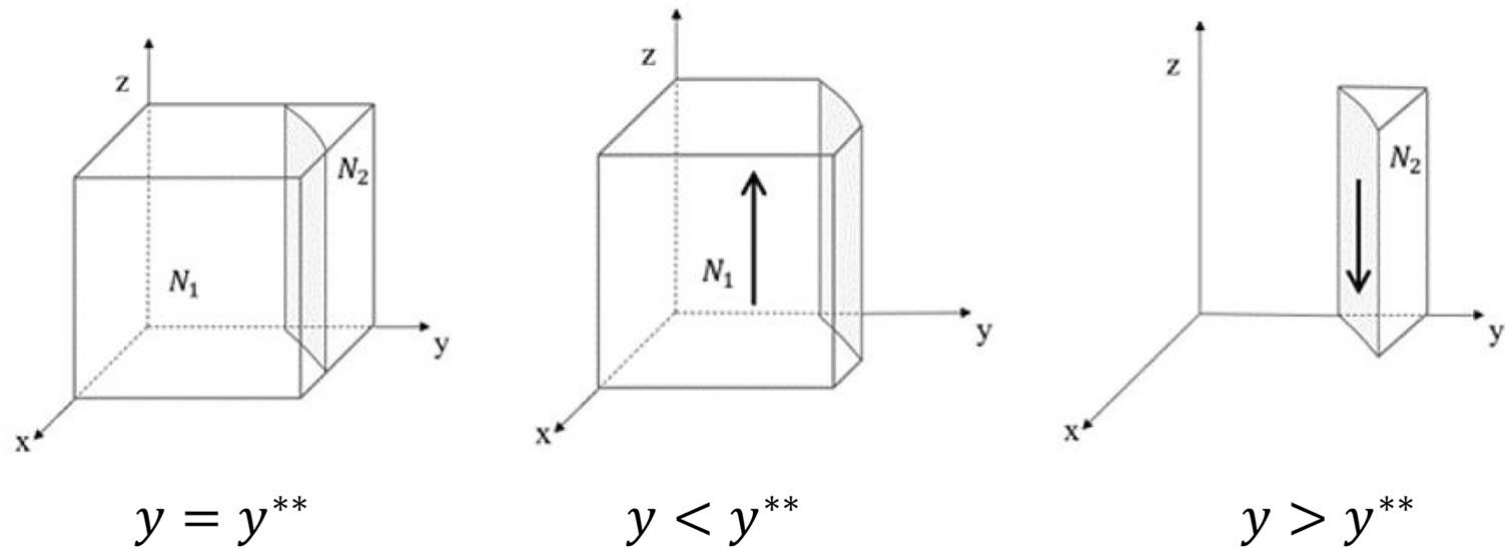
Phase diagram of government strategy evolution.

The evolutionary phase diagram of government’s strategy choice is shown in Fig. 4.

From Fig. 4, it is known that the volume of part ${N}_{1}$ is the probability of government choosing the strategy of “strict supervision”, and we use ${V}_{{N}_{1}}$ to represent it. Meanwhile, the volume of part ${N}_{2}$ is the probability of government choosing the strategy of “loose supervision”, and we use ${V}_{{N}_{2}}$ to represent it. From the formula, we can get:

23 $${V}_{{N}_{1}}={\int }_{0}^{1}{\int }_{0}^{1}\frac{{H}_{4}-{R}_{2}-{K}_{1}-{K}_{2}-{H}_{5}-T-\alpha {P}_{2}-x\left(\alpha {P}_{1}-{\alpha {P}_{2}-K}_{2}\right)}{x\left(B+\alpha {P}_{2}-\alpha {P}_{1}\right)-B-\alpha {P}_{2}-{K}_{1}-T}dxdz$$

24 $${V}_{{N}_{2}}=1-{V}_{{N}_{1}}$$

*Inference 5* The probability that the government chooses the strategy of "strict supervision" is positively correlated with respect to K_2_, T, and P_2_, and negatively correlated with respect to B.

From Eq. (23), we can get:

$$\frac{\partial {V}_{{N}_{1}}}{\partial {K}_{2}}>0,\frac{\partial {V}_{{N}_{1}}}{\partial T}>0,\frac{\partial {V}_{{N}_{1}}}{\partial {P}_{2}}>0,\frac{\partial {V}_{{N}_{1}}}{\partial B}<0$$

From the above partial derivative results, it is clear that the higher the amount of punishment set by the government, the more it will encourage the government to strictly supervise the process of medical supplies allocation. And increasing the number of incentives set will reduce the probability of government choosing the strategy of “strict supervision”. In addition, the greater the loss to the government due to accountability by the higher authorities, the more it will encourage the government to strictly fulfill the responsibility of strict supervision.

*Inference 6* In the process of a tripartite evolutionary game, as the probability of GNPO choosing the strategy of “able to allocate on demand” decreases or the probability of the hospital choosing the strategy of “accept” decreases, the probability of government choosing the strategy of “strict supervision” increases.

From the strategy stability analysis of government, it is known that when $${y<y}^{**}$$, $$\mathrm{M}\left(y\right)<0$$. And $$\mathrm{z}=1$$ is the evolutionary stability strategy of government. When $${y>y}^{**}$$, $$\mathrm{M}\left(y\right)<0$$. And z $$=0$$ is the evolutionary stability strategy of the government. In addition, because $${y}^{**}=\frac{{H}_{4}-{R}_{2}-{K}_{1}-{K}_{2}-{H}_{5}-T-\mathrm{\alpha }{P}_{2}-x\left(\mathrm{\alpha }{P}_{1}-{\mathrm{\alpha }{P}_{2}-K}_{2}\right)}{x\left(B+\mathrm{\alpha }{P}_{2}-\mathrm{\alpha }{P}_{1}\right)-\mathrm{B}-\mathrm{\alpha }{P}_{2}-{K}_{1}-T}$$, the probability that government’s stabilization strategy is $$\mathrm{z}=1$$ will be improved with the gradual decrease of x or y.

The above analysis illustrates that the probability of strict supervision by government is influenced by the strategies of GNPO and hospital. When the GNPO tends to choose the strategy of “unable to allocate on demand” or the hospital tends to choose the strategy of “refuse and fight”, the government will increase the probability of strict supervision and the intensity of supervision. That will lead to a more effective and scientific flow of medical supplies, thus optimizing the measures of battling epidemic and improving the level of epidemic prevention and control.

### Stability analysis of the equilibrium point of tripartite evolutionary game system

From the stability principle of the differential equation, when the strategy is in a state of stable equilibrium, we have F(x) = 0, F(y) = 0, and F(z) = 0. The game equilibrium point can be derived from the following equation:

25 $$\left\{ {\begin{array}{*{20}l} {F\left( x \right) = \frac{dx}{{dt}} = x(E_{11} - E_{1} ) = x\left( {1 - x} \right)\left[ {y\left( {\alpha V_{1} - \alpha V_{2} } \right) + \alpha V_{2} - \alpha V_{1} + zK_{2} - A} \right] = 0} \hfill \\ {F\left( y \right) = \frac{dy}{{dt}} = y(E_{21} - E_{2} ) = y\left( {1 - y} \right)\left[ {z\left( {x*\alpha P_{1} - x*\alpha P_{2} - xB + B + \alpha P_{2} } \right) + x\left( {\alpha W_{2} - \alpha W_{1} } \right) + \alpha H_{2} - \alpha W_{2} } \right] = 0} \hfill \\ {F\left( z \right) = \frac{dz}{{dt}} = z(E_{31} - E_{3} ) = z\left( {1 - z} \right)\left[ {R_{2} + K_{1} + K_{2} - H_{4} + H_{5} + T + \alpha P_{2} + x\left( {\alpha P_{1} - \alpha P_{2} - K_{2} } \right) + y\left( { - B - \alpha P_{2} - K_{1} - T} \right) + xy\left( {B - \alpha P_{1} + \alpha P_{2} } \right)} \right] = 0} \hfill \\ \end{array} } \right.$$

In an asymmetric game, if the evolutionary game equilibrium E is an evolutionary stable equilibrium, then E must be a strict Nash equilibrium. And a strict Nash equilibrium is a pure strategy equilibrium^56^. That is, a mixed strategy equilibrium in an asymmetric game must not be an evolutionary stable equilibrium. Therefore, in this paper, we only need to discuss the asymptotic stability of the pure strategy equilibrium point, and do not need to consider the mixed strategy solutions. From Eq. (25), we can know that the eight pure-strategic Nash equilibrium points are E_1_ (0, 0, 0), E_2_ (1, 0, 0), E_3_ (0, 1, 0), E_4_ (0, 0, 1), E_5_ (1, 1, 0), E_6_ (1, 0, 1), E_7_ (0, 1, 1), E_8_ (1, 1, 1). Then we find the first-order partial derivatives of F(x), F(y), and F(z) with respect to x, y, and z, respectively. The Jacobi matrix of this tripartite game system is obtained as

$$J=\left[\begin{array}{ccc}\frac{\partial F\left(x\right)}{\partial x}& \frac{\partial F\left(x\right)}{\partial y}& \frac{\partial F\left(x\right)}{\partial z}\\ \frac{\partial F\left(y\right)}{\partial x}& \frac{\partial F\left(y\right)}{\partial y}& \frac{\partial F\left(y\right)}{\partial z}\\ \frac{\partial F\left(z\right)}{\partial x}& \frac{\partial F\left(z\right)}{\partial y}& \frac{\partial F\left(z\right)}{\partial z}\end{array}\right]=\left[\begin{array}{ccc}{a}_{11}& {a}_{12}& {a}_{13}\\ {a}_{21}& {a}_{22}& {a}_{23}\\ {a}_{31}& {a}_{32}& {a}_{33}\end{array}\right]$$

where:

$${a}_{11}=(x-1)(A+\alpha {V}_{1}-\alpha {V}_{2}-z{K}_{2}-\alpha y{V}_{1}+\alpha y{V}_{2})+x(A+\alpha {V}_{1}-\alpha {V}_{2}-z{K}_{2}-\alpha y{V}_{1}+\alpha y{V}_{2})$$

$${a}_{12}=x(1-x)(\alpha {V}_{1}-\alpha {V}_{2})$$

$${a}_{13}=x(x-1)(-{K}_{2})$$

$${a}_{21}=y(y-1)({\alpha W}_{1}-{\alpha W}_{2}+zB-\alpha z{P}_{1}+\alpha z{P}_{2})$$

$${a}_{22}=(1-y)({\alpha H}_{2}-{\alpha W}_{2}+zB+\alpha z{P}_{2}-{\alpha xW}_{1}+{\alpha xW}_{2}-zxB+\alpha xz{P}_{1}-\alpha xz{P}_{2})-y({\alpha H}_{2}-{\alpha W}_{2}+zB+\alpha z{P}_{2}-{\alpha xW}_{1}+{\alpha xW}_{2}-zxB+\alpha xz{P}_{1}-\alpha xz{P}_{2})$$

$${a}_{23}=y(1-y)(B+\alpha {P}_{2}-xB+\alpha x{P}_{1}-\alpha x{P}_{2})$$

$${a}_{31}=z(z-1)({K}_{2}-\alpha {P}_{1}+\alpha {P}_{2}-yB+\alpha y{P}_{1}-\alpha y{P}_{2})$$

$${a}_{32}=z(z-1)(B+{K}_{1}+T+\alpha {P}_{2}-xB+\alpha x{P}_{1}-\alpha x{P}_{2})$$

$${a}_{33}=(-z)({H}_{5}-{H}_{4}+{K}_{1}+{K}_{2}+{R}_{2}+T+\alpha {P}_{2}-yB-x{K}_{2}-y{K}_{1}-yT+\alpha x{P}_{1}-\alpha x{P}_{2}-\alpha y{P}_{2}+xyB-\alpha x{yP}_{1}+\alpha xy{P}_{2})-(z-1)({H}_{5}-{H}_{4}+{K}_{1}+{K}_{2}+{R}_{2}+T+\alpha {P}_{2}-yB-x{K}_{2}-y{K}_{1}-yT+\alpha x{P}_{1}-\alpha x{P}_{2}-\alpha y{P}_{2}+xyB-\alpha x{yP}_{1}+\alpha xy{P}_{2})$$

By substituting the calculated equilibrium points E_1_–E_8_ into the above Jacobi matrix, the Jacobi matrix corresponding to each point can be obtained respectively. In the example of E_1_ (0, 0, 0), the Jacobi matrix corresponding to this point is

$${J}_{{E}_{1}}=\left[\begin{array}{ccc}{\alpha V}_{2}-{\alpha V}_{1}-A& 0& 0\\ 0& {\alpha H}_{2}-{\alpha W}_{2}& 0\\ 0& 0& {H}_{5}-{H}_{4}+{K}_{1}+{K}_{2}+{R}_{2}+T+\alpha {P}_{2}\end{array}\right]$$

The three eigenvalues of the matrix ${J}_{{E}_{1}}$ are obtained as

$${{\lambda }_{1}=\,}{\alpha V}_{2}-{\alpha V}_{1}-A, {{\lambda }_{2}=}{\alpha H}_{2}-{\alpha W}_{2}, {{\lambda }_{3}=H}_{5}-{H}_{4}+{K}_{1}+{K}_{2}+{R}_{2}+T+\alpha {P}_{2}$$

The Lyapunov indirect method is a basic method for analyzing the stability of differential equations in modern cybernetics, and it is widely applied in system stability analysis. For example, Lu et al. adopted the Lyapunov indirect method to analyze the stability problem of a two-armed robot teleoperating system^57^. However, it is rarely applied to the stability analysis of tripartite evolutionary game systems.

In this paper, we adopt Lyapunov’s discriminant method (indirect method) to determine the asymptotic stability of the game equilibrium point^58^. Thus, there are the following conclusions.

1. If all the eigenvalues of the Jacobi matrix have negative real parts, then the equilibrium point is an asymptotically stable point.
2. If at least one of the eigenvalues of the Jacobi matrix has a positive real part, then the equilibrium point is an unstable point.
3. If the eigenvalues of the Jacobi matrix have negative real parts except for the eigenvalues that have a real part of zero, the equilibrium point is in a critical state, and the stability cannot be determined by the sign of the eigenvalues.

The stability of the above eight game equilibrium points is shown in Table 3.

**Table 3:** Equilibrium point stability analysis.

Equilibrium point	Eigenvalues of Jacobian matrix			The symbol of real part	Stability	Condition
	λ_1_	λ_2_	λ_3_			
E_1_ (0, 0, 0)	${\alpha V}_{2}-{\alpha V}_{1}-A$	${\alpha H}_{2}-{\alpha W}_{2}$	${H}_{5}-{H}_{4}+{K}_{1}+{K}_{2} +{R}_{2}+T+\alpha {P}_{2}$	(× , −, +)	Unstable point	–
E_2_ (1, 0, 0)	${\alpha H}_{2}-{\alpha W}_{1}$	$A+{\alpha V}_{1}-{\alpha V}_{2}$	${H}_{5}-{H}_{4}+{K}_{1}+{R}_{2}+T+\alpha {P}_{1}$	(−, × , +)	Unstable point	–
E_3_ (0, 1, 0)	${\alpha W}_{2}-{\alpha H}_{2}$	$-A$	${H}_{5}-{H}_{4}-B+{K}_{2}+{R}_{2}$	(−, −, −)	ESS	(1)
E_4_ (0, 0, 1)	${K}_{2}-A-{\alpha V}_{1}+{\alpha V}_{2}$	$B+\alpha {H}_{2}+\alpha {P}_{2}-{\alpha W}_{2}$	${H}_{4}{-H}_{5}-{K}_{1}-{K}_{2}$ $-{R}_{2}-T-\alpha {P}_{2}$	(+ , + , −)	Unstable point	–
E_5_ (1, 1, 0)	$A$	${\alpha W}_{1}-{\alpha H}_{2}$	${H}_{5}-{H}_{4}+{R}_{2}$	(+ , + , ×)	Unstable point	–
E_6_ (1, 0, 1)	${{\alpha H}_{2}+\alpha {P}_{1}-\alpha W}_{1}$	$A-{K}_{2}+{\alpha V}_{1}-{\alpha V}_{2}$	${H}_{4}{-H}_{5}-{K}_{1}-{R}_{2}-T-\alpha {P}_{1}$	(+ , −, −)	Unstable point	–
E_7_ (0, 1, 1)	${K}_{2}-A$	${\alpha W}_{2}-{\alpha H}_{2}-\alpha {P}_{2}-B$	${B+{H}_{4}-H}_{5}-{K}_{2}-{R}_{2}$	(+ , −, ×)	Unstable point	–
E_8_ (1, 1, 1)	$A-{K}_{2}$	${{H}_{4}-H}_{5}-{R}_{2}$	${\alpha W}_{1}-\alpha {P}_{1}-{\alpha H}_{2}$	(−, −, −)	ESS	(2)

“ + ” means the symbol of the real part is positive, “−” means the symbol of the real part is negative, and “×” means the symbol of the real part is uncertain.

(1) $${\alpha W}_{2}-{\alpha H}_{2}<0$$,$${H}_{5}-{H}_{4}-B+{K}_{2}+{R}_{2}<0$$; (2) $$A-{K}_{2}<0$$, $${{H}_{4}-H}_{5}-{R}_{2}<0$$, $${\alpha W}_{1}-\alpha {P}_{1}-{\alpha H}_{2}<0$$

*Inference 7:* When conditions (1) and (2) are satisfied, there are two equilibrium stabilization points at E_3_ (0, 1, 0) and E_8_ (1, 1, 1) in the replicated dynamic system.

Inference 7 shows that the GNPO chooses the strategy of “unable to allocate on demand”, the hospital chooses the strategy of “accept”, and the government chooses the strategy of “loose supervision” is a stable set of strategies. GNPO chooses the strategy of “able to allocate on demand”. The hospital chooses the strategy of “accept”, and the government chooses the strategy of “strict supervision” is also a stable set of strategies.

From condition (1), it can be seen that when the government chooses the strategy of “loose supervision”, the government has no information about the strategy choices of GNPO and the hospital, and at this time, the government does not reward or punish the behavior of GNPO and the hospital. Meanwhile, the additional supplementary benefit the hospital gets from GNPO is less than its own cost of fighting. Therefore, if the GNPO chooses the strategy of “unable to allocate on demand” at this time, the hospital chooses to accept the medical supplies allocation plan in its own interest. At this point, the strategy combination evolves to be stable (unable to allocate on demand, accept, loose supervision).

From condition (2), we can know that in the emergency time of epidemic prevention and control, the value of the difference between the cost of strict supervision and its benefits such as enhanced social credibility, is less than the cost of loose supervision for the government, so it is in the government’s self-interest to choose the strategy of “strict supervision” at this time. Meanwhile, the additional benefits of choosing the strategy of “unable to allocate on demand” for the GNPO are less than the punishment of the government, so it is in the GNPO’s own interest to choose reasonable allocation of medical supplies. For the hospital, the difference in value between the additional benefit and the cost of fighting due to choosing the strategy of “refuse and fight” is less than the government’s punishment at this time, so it is in the hospital’s self-interest to choose the strategy of “accept”. At this point, the strategy combination has evolved to stabilize at (able to allocate on demand, accept, strict supervision). Such a combination of strategies meets the interests of all three players. At the same time, it satisfies the maximization of the overall social benefits and is conducive to quickly blocking the spread of the virus and winning the battle against the epidemic.

## Numerical simulation

### Computational case

COVID-19, which is being experienced by people worldwide, is a major public health emergency. Since the outbreak of COVID-19 at the end of 2019, hundreds of millions of people have been infected worldwide, posing a great risk to human lives and also leading to a global economic recession. As the main front in the fight against COVID-19, hospitals have been hit very hard by the epidemic. In 2020, when Wuhan City was the hardest hit by the epidemic, the government urgently requisitioned more than sixty hospitals as “fever clinics” to provide centralized treatment for infected patients^22^. At this time, although the GNPO, which was designated by the government as the sole distributor of socially donated medical supplies, had received a large amount of medical supplies from the community. There was still an extreme shortage of medical supplies and a large gap in the demand for medical supplies from medical staff and patients.

In this case, the Union Hospital of Tongji Medical College of Huazhong University of Science and Technology, an important designated hospital, received only 3000 medical surgical masks from the GNPO, but Wuhan Renai Hospital, which did not receive infected patients at this time, received 16,000 N95 masks from the GNPO. This situation had also raised doubts in the community about the rationality of the allocation process of medical supplies by GNPOs in COVID-19. On the one hand, GNPOs such as the Red Cross Society of China Hubei Branch were unable to coordinate and allocate the large amount of medical supplies that were suddenly increased due to a lack of adequate staff and corresponding technical skills. And on the other hand, due to the shock of the sudden arrival of COVID-19, the lack of effective supervision of the resource allocation process by governmental regulatory authorities also led to the irrationality of the allocation process.

Therefore, this paper takes the allocation process of medical supplies by GNPOs in Wuhan during COVID-19 as a case study to provide a more scientific reference for the actual problem. Under this situation, the study focuses on the sensitivity of the game players’ strategies to the model parameters so that the randomness of the parameter values does not affect the results of the simulation^59^.

When the epidemic strikes, the hospitals are the first to be hit and suffer losses (denoted as E) from the epidemic. If the GNPO chooses the strategy of “unable to allocate on demand” at this time, it is likely that the hospital will suffer additional losses (denoted as D). Generally, inpatient revenue accounts for about 50% of the total hospital’s revenue, and outpatient volume decreases significantly when the epidemic strikes. Therefore, the reduction in the total hospital’s revenue during an epidemic can be approximated as the reduction in inpatient medical revenue. We set the initial value of parameter D to 300 and the initial value of parameter E to 200 with reference to existing studies^60,61^.

A review of existing literature shows that the government that chooses the strategy of supervision can reduce the occurrence of unscientific behavior during an epidemic outbreak and greatly strengthen the confidence of the public in fighting the epidemic. It is very important for the government to enhance its credibility and win the battle against the epidemic. However, the GNPO, as the allocator of medical supplies, may cause an unscientific allocation of medical supplies due to the imperfection of the supplies allocation system. At this time, if the hospital, as the receiver of the supplies, chooses the strategy of “refuse and fight”, the government will take certain punitive measures against both sides for the sake of the overall situation of battling the epidemic and social stability. In this paper, the parameter $\mathrm{\alpha }$ is a value between 0 and 1, and we set the initial value of $\mathrm{\alpha }$ to 0.6. Combining the relevant literature and the real situation in China, we set the initial parameters of each variable in the tripartite game process as: K_1_ = 80, K_2_ = 60, V_1_ = 100, V_2_ = 150, A = 25, H_1_ = 80, D = 300, E = 200, α = 0.6, W_1_ = 80, W_2_ = 100, B = 80, P_1_ = 160, P_2_ = 80, H_2_ = 110, R_1_ = 170, H_3_ = 160, H_4_ = 100, H_5_ = 50, R_2_ = 60, T = 150.And through PYTHON software, we investigate the influencing factors of different behavioral strategies among GNPO, hospital, and the government under different parameters^40,42,44,62,63^.

### Parameter sensitivity analysis

**Figure 5:**
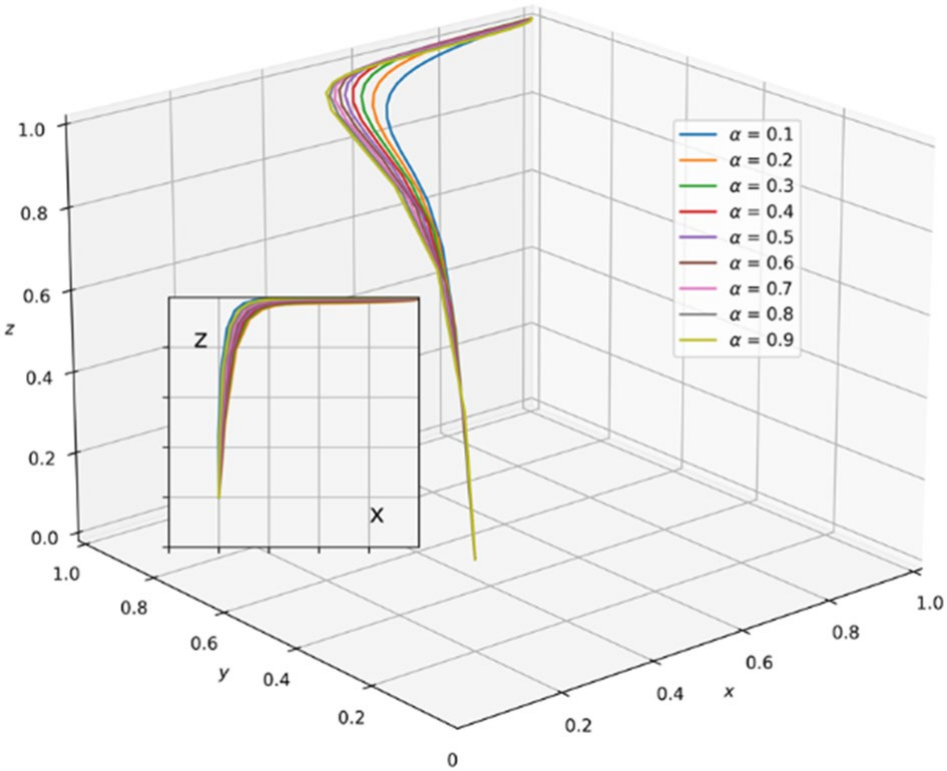
Effect of $\mathrm{\alpha }$ on the equilibrium outcome.

*Impact of α* To analyze the effect of $\mathrm{\alpha }$ on the evolutionary game process and outcome, we vary the value of $\mathrm{\alpha }$ from 0.1 to 0.9 sequentially. The simulation results of replicating dynamic equations evolving over time are shown in Fig. 5.

From Fig. 5, we can draw the following conclusions during the evolution of the system to the stabilization point. As $\mathrm{\alpha }$ increases, the probability of GNPO choosing the strategy of “able to allocate on demand” decreases, and the probability of the government choosing the strategy of “strict supervision” increases, and will gradually increase to 1. This indicates that as $\mathrm{\alpha }$ increases, the GNPO will be more inclined to choose unscientific allocation of medical supplies, and the government will be more inclined to choose strict supervision of the allocation process. Therefore, when the government strictly supervises the allocation process of medical supplies, the hospitals should consider the overall situation of battling against an epidemic and reasonably reduce their willingness to not accept the allocation plan of medical supplies. That will help the GNPO make the allocation plan of medical supplies more scientifically.

**Figure 6:**
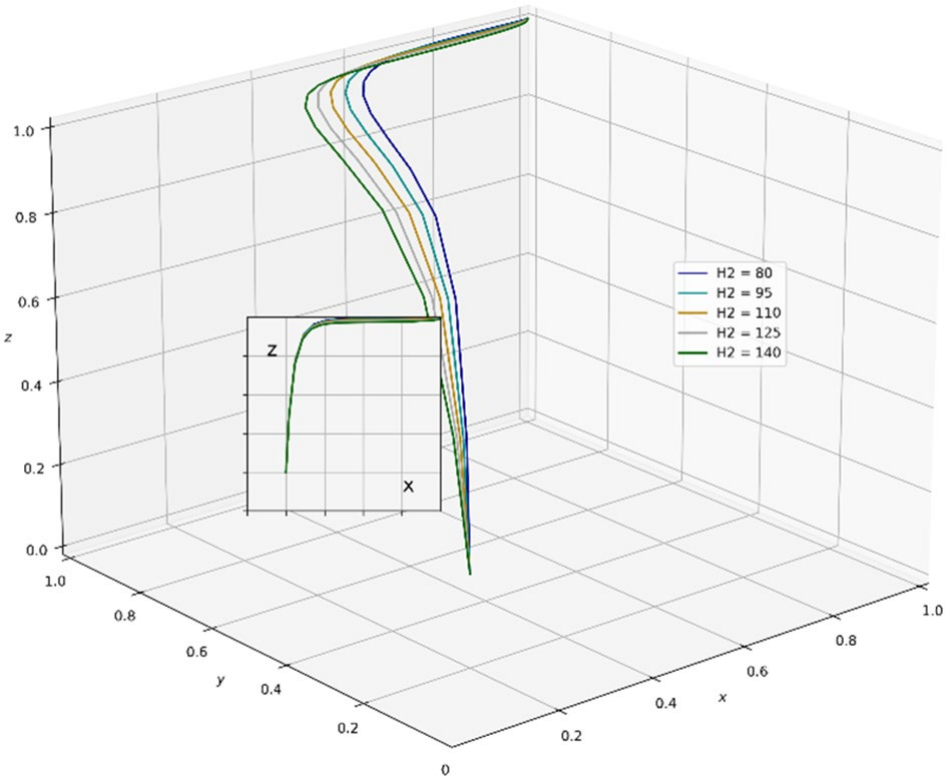
Effect of H_2_ on the equilibrium outcome.

*Impact of H*_*2*_. To analyze the effect of H_2_ on the evolutionary game process and outcome, we assign H_2_ = 80, 95, 110, 125, 140, respectively. The simulation results of replicating dynamic equations evolving over time are shown in Fig. 6.

From Fig. 6, we can draw the following conclusions during the evolution of the system to the stabilization point. As H_2_ increases, the probability of GNPO choosing the strategy of “able to allocate on demand” increases, and will gradually increase to 1, while the probability of the government choosing the strategy of “strict supervision” decreases. This indicates that as H_2_ increases, the GNPO will be more inclined to choose scientific allocation of medical supplies, and the government will be more inclined to choose loose supervision of the allocation process. Therefore, when the government strictly supervises the medical supplies allocation process, it can appropriately increase the coordination costs incurred due to the hospitals’ fighting. That will help the GNPO allocate medical supplies more scientifically.

**Figure 7:**
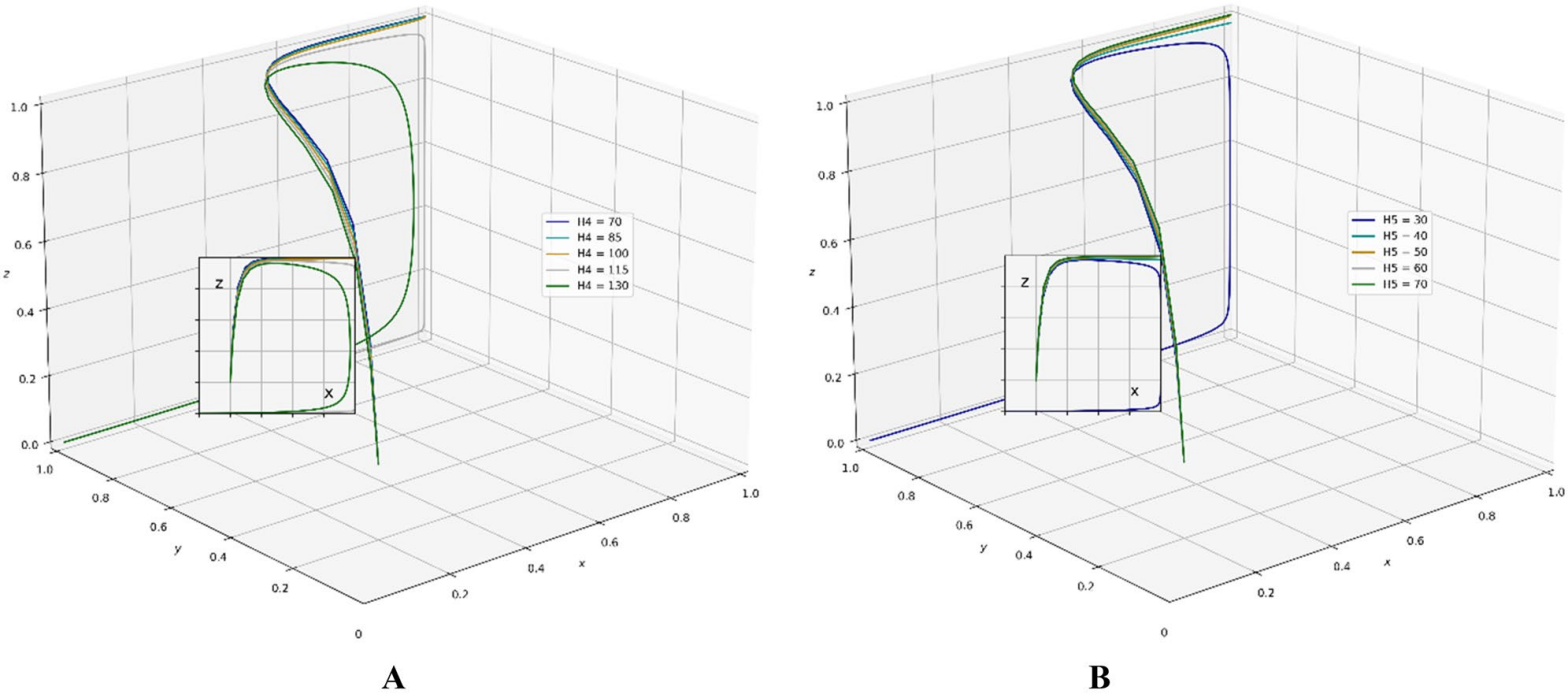
Effect of the cost of government supervision on the equilibrium outcome. (**A**) Effect of H_4_ on the equilibrium outcome. (**B**) Effect of H_5_ on the equilibrium outcome.

*Impact of H*_*4*_*.* To analyze the effect of H_4_ on the evolutionary game process and outcome, we assign H_4_ = 70, 85, 100, 115, 130, respectively. The simulation results of replicating dynamic equations evolving over time are shown in Fig. 7A.

From Fig. 7A, we can draw the following conclusions during the evolution of the system to the stabilization point. As H_4_ increases, the probability of GNPO choosing the strategy of “able to allocate on demand” increases, and will gradually increase to 1, while the probability of the government choosing the strategy of “strict supervision” decreases. This indicates that as the cost of strict government supervision increases, GNPO will be more inclined to choose scientific allocation of medical supplies, and the government will be more inclined to choose loose supervision of the allocation process at this time. It is interesting to observe that when the cost of strict government supervision increases to a certain threshold, the GNPO will tend to choose the strategy of “unable to allocate on demand” and the government will tend to choose loose regulation. At this point, the hospital will still choose the strategy of “Accept”. This is because when the strict supervision cost of the government exceeds its own acceptable range, the government with bounded rationality will tend to choose the strategy of “Loose supervision” in order to maximize its own interests. In this case, GNPO, under the pressure of ultra-strict supervision, has an incentive to choose the strategy of “Unable to allocate on demand” to ensure that their own interests are maximized. However, since the government is unable to provide incentives and penalties under the strategy of loose supervision, the hospitals can only choose the strategy of “Accept” to ensure their own interests are maximized. Therefore, the increased cost of strict government supervision helps GNPOs allocate medical supplies more scientifically. However, GNPOs will allocate medical supplies unscientifically under heavy pressure when the cost of supervision exceeds the threshold.

*Impact of H*_*5*_ To analyze the effect of H_5_ on the evolutionary game process and outcome, we assign H_5_ = 30, 40, 50, 60, 70, respectively. The simulation results of replicating dynamic equations evolving over time are shown in Fig. 7B.

From Fig. 7B, we can draw the following conclusions during the evolution of the system to the stabilization point. As H_5_ increases, the probability of GNPO choosing the strategy of “able to allocate on demand” decreases, and the probability of the government choosing the strategy of “strict supervision” increases. This indicates that as the cost of loose government supervision increases, the GNPO will be more inclined to choose unscientific allocation of medical supplies, and the government will be more inclined to choose strict supervision of the allocation process at this time. It is also interesting to observe that when the cost of loose government supervision is less than a certain threshold, the GNPO will tend to choose the strategy of “unable to allocate on demand” and the government will tend to choose loose supervision. Therefore, GNPOs may allocate medical supplies unscientifically when the government loosely supervises the allocation process of medical supplies. In addition, elevating the cost of loose government supervision helps the government choose to strictly supervise the allocation process of medical supplies.

**Figure 8:**
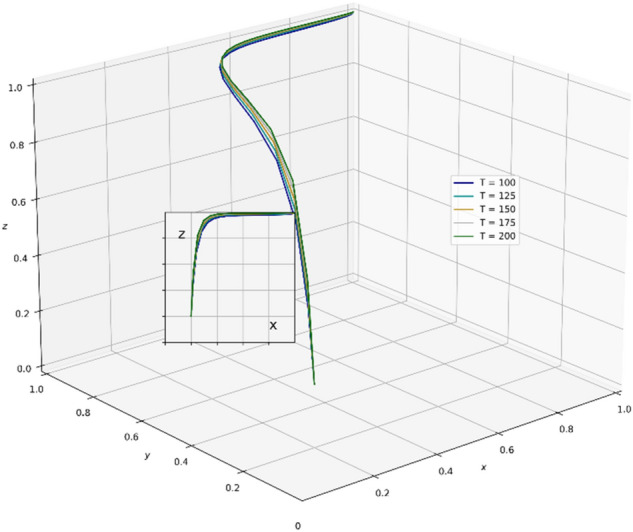
Effect of T on the equilibrium outcome.

*Impact of T* To analyze the effect of T on the evolutionary game process and outcome, we assign T = 100, 125, 150, 175, 200, respectively. The simulation results of replicating dynamic equations evolving over time are shown in Fig. 8.

From Fig. 8, we can draw the following conclusions during the evolution of the system to the stabilization point. As T increases, the probability of GNPO choosing the strategy of “able to allocate on demand” decreases, and the probability of the government choosing the strategy of “strict supervision” increases. This indicates that as the loss to the government due to the accountability of higher authorities increases, the GNPO will be more inclined to choose unscientific allocation of medical supplies, and the government will be more inclined to choose strict supervision of the allocation process at this time. Therefore, when the government is loose in its supervision, it will be held accountable by higher authorities because of the serious negative impact of the hospital’s fighting on social stability. In addition, elevating the loss to the government due to the higher authority’s accountability helps the government choose to strictly supervise the allocation process of medical supplies.

**Figure 9:**
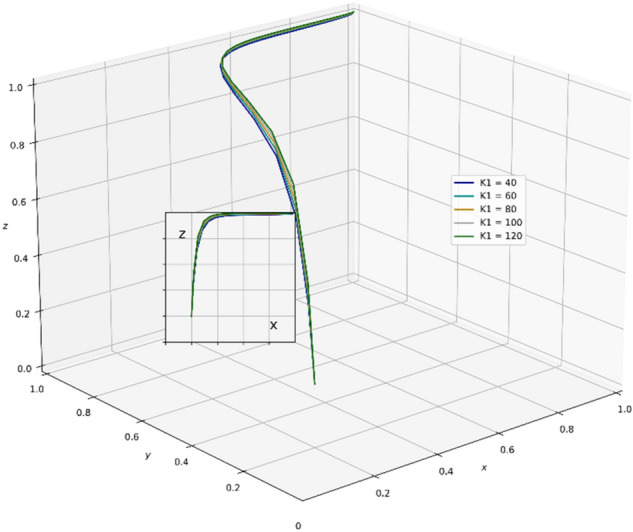
Effect of K_1_ on the equilibrium outcome.

*Impact of K*_*1*_ To analyze the effect of K_1_ on the evolutionary game process and outcome, we assign K_1_ = 40, 60, 80, 100, 120, respectively. The simulation results of replicating dynamic equations evolving over time are shown in Fig. 9.

From Fig. 9, we can draw the following conclusions during the evolution of the system to the stabilization point. As K_1_ increases, the probability of GNPO choosing the strategy of “able to allocate on demand” decreases, and the probability of the government choosing the strategy of “strict supervision” increases. This indicates that when the government strictly supervises, as the punishment given to GNPO due to the fighting of hospital increases, GNPO will be more inclined to choose to allocate medical supplies unscientifically, and the government will be more inclined to choose to strictly supervise the allocation process of medical supplies. When the government strictly supervises the allocation process, if it simply increases the punishment given to GNPO due to the fighting of hospital, GNPO may allocate the medical supplies unscientifically under heavy pressure. Therefore, the government needs to formulate a more scientific punishment mechanism to make the allocation of medical supplies more efficient.

**Figure 10:**
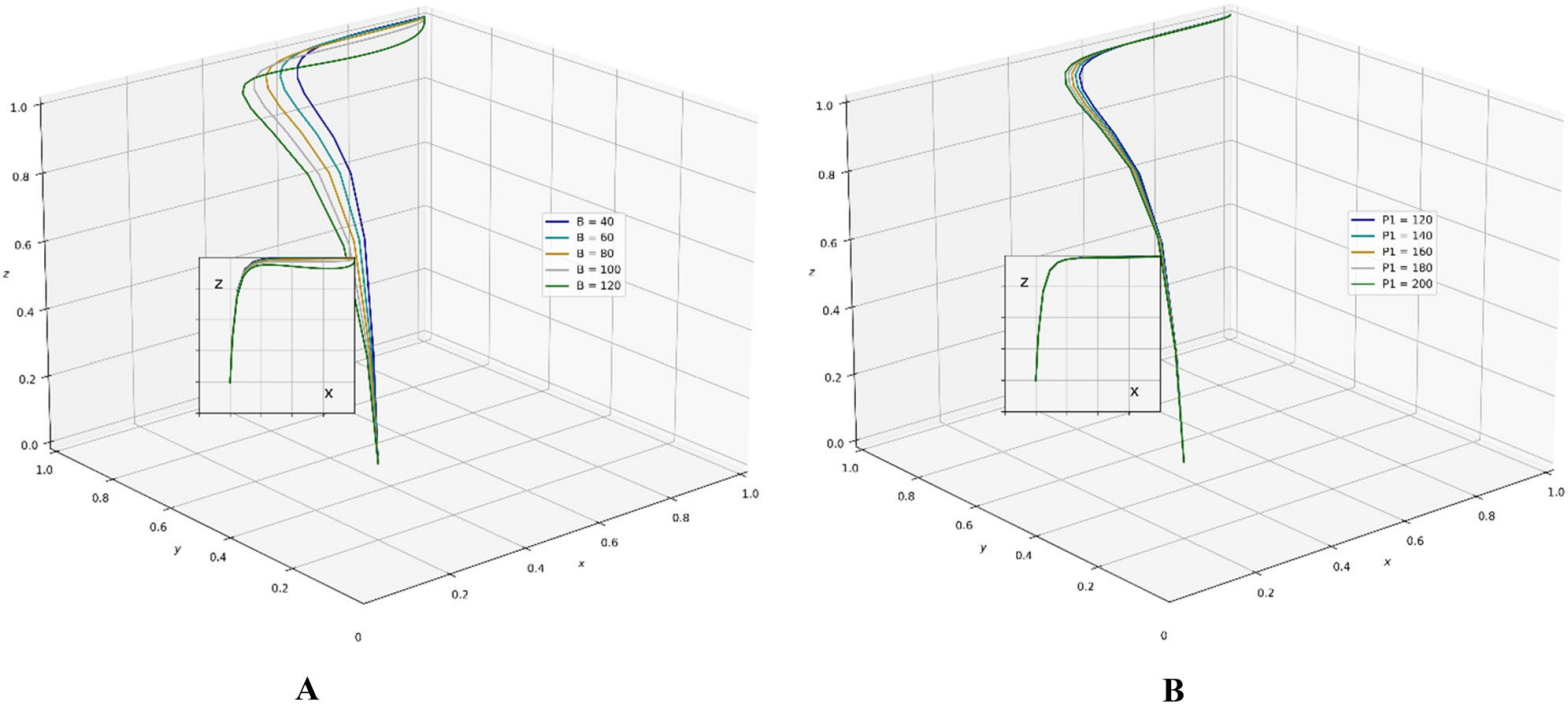
(**A**) Effect of B on the equilibrium outcome. (**B**) Effect of P_1_ on the equilibrium outcome.

*Impact of B* To analyze the effect of B on the evolutionary game process and outcome, we assign B = 40, 60, 80, 100, 120, respectively. The simulation results of replicating dynamic equations evolving over time are shown in Fig. 10A.

From Fig. 10A, we can draw the following conclusions during the evolution of the system to the stabilization point. As B increases, the probability of GNPO choosing the strategy of “able to allocate on demand” increases, and the probability of the government choosing the strategy of “strict supervision” decreases. This indicates that when the hospital accepts the GNPO’s unscientific supplies allocation plan, the GNPO will be more inclined to choose to allocate medical supplies scientifically, and the government will be more inclined to choose to loosely supervise the allocation process of medical supplies as the incentives given to the hospital by the government increase. Therefore, the increase in incentives given to the hospital by the government will reduce the hospital’s willingness to fight against the GNPO’s allocation plan if the hospital accepts the GNPO’s unscientific supplies allocation plan. That will enable the GNPO to better control the overall allocation of medical supplies and make a more scientific allocation plan. Meanwhile, the government would be inclined to choose to loosely supervise the allocation process because of the reduction in fighting. This is good for the overall situation of epidemic prevention and control.

*Impact of P*_*1*_ To analyze the effect of P_1_ on the evolutionary game process and outcome, we assign P_1_ = 120, 140, 160, 180, 200, respectively. The simulation results of replicating dynamic equations evolving over time are shown in Fig. 10B. From Fig. 10B, we can know that the impact of P_1_ on the equilibrium outcome is similar to that of B.

**Figure 11:**
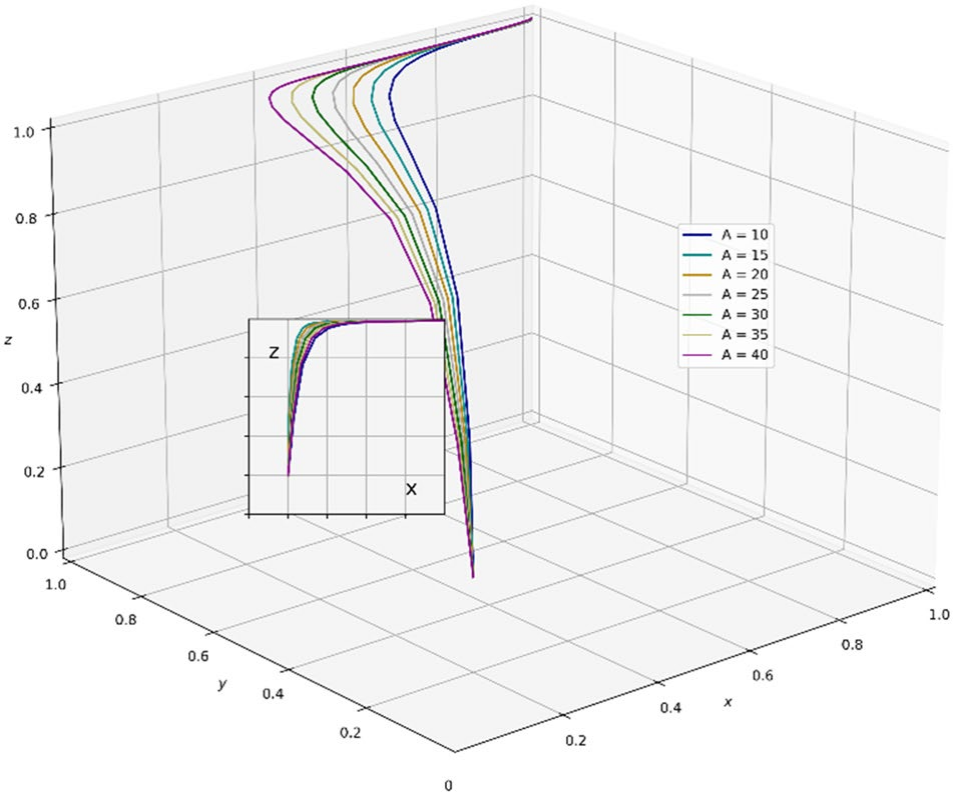
Effect of A on the equilibrium outcome.

*Impact of A* To analyze the effect of A on the evolutionary game process and outcome, we assign A = 10, 15, 20, 25, 30, 35, 40, respectively. The simulation results of replicating dynamic equations evolving over time are shown in Fig. 11.

From Fig. 11, we can draw the following conclusions during the evolution of the system to the stabilization point. When A is not greater than 25, as A increases, the probability that GNPO chooses the strategy of “able to allocate on demand” decreases and the probability that the government chooses the strategy of “strict supervision” increases. When A is not less than 25, as A increases, the probability that GNPO chooses the strategy of “able to allocate on demand” increases, and the probability that the government chooses the strategy of “strict supervision” decreases. This indicates that when the additional benefit to GNPO is less than a certain threshold due to opportunistic behavior such as an unscientific supplies allocation plan, as the benefit increases, GNPO will be more inclined to choose to allocate medical supplies unscientifically, and the government will be more inclined to choose to strictly supervise the allocation process of medical supplies. When the additional benefit to GNPO is greater than this value due to opportunistic behavior, as the benefit increases, GNPO will prefer to allocate medical supplies scientifically, and the government will prefer to loosely supervise the allocation process of medical supplies. It may be due to the fact that the additional benefits received by the GNPO have attracted the attention of society or that the government has increased its supervision. The greater the additional benefit, the greater the motivation for GNPO to abandon the scientific allocation plan.

Therefore, a more comprehensive review of the reasonableness of the allocation plan can help the GNPO allocate medical supplies in a more scientific manner. In addition, the government can reduce the additional benefits that GNPO can gain through opportunistic behavior, such as unscientific allocation plans, and improve the probability of its own strict supervision. That will help GNPO allocate medical supplies more scientifically.

## Discussions

This paper invokes the government supervision mechanism and constructs a tripartite evolutionary game model of GNPO-hospital-government to investigate the allocation process of medical supplies in the rescue environment of public health emergencies under incomplete information. Under the assumption of “bounded rationality”, the stability of the strategy choices of the three game players and the stability of the equilibrium strategy combination of the game system are analyzed. At the same time, this paper discusses the impact of the changes to the parameters related to the replicating dynamic equation on the equilibrium strategies of the game players using the quantitative analysis method. The main suggestions are shown below.

Firstly, as the receiver of medical supplies, the hospital should consider the overall situation of battling against the epidemic and reasonably decrease its willingness to not accept the allocation plan of medical supplies. It will help the GNPO allocate medical supplies more scientifically, so that medical supplies can be circulated in a rational and orderly manner in the case of public health emergencies. Only when medical supplies are scientifically circulating can the hospitals have enough weapons to fight the epidemic and restore their own medical treatment orders as soon as possible. That is of great significance in reducing the risk of infection among health care workers during public health emergencies.

Secondly, the GNPO should defuse the fighting of the hospital and maintain social stability before it breaks out on a large scale. Increasing incentives for the hospital due to accepting the GNPO’s allocation plan when the GNPO chooses the strategy of “unable to allocate on demand” under strict government supervision, increasing punishment for the hospital due to its strategy of “refuse and fight” when the GNPO chooses the strategy of “able to allocate on demand” under strict government supervision, and decreasing punishment for the GNPO due to the hospital’s strategy of “refuse and fight” under strict government supervision would all help the GNPO to allocate medical supplies more scientifically, but may not help the government take its responsibility for strict supervision more seriously at this time. The government needs to set up a reasonable reward and punishment mechanism that must satisfy the condition that the sum of rewards and punishments for all stakeholders is greater than their speculative gains. That will guarantee a rational and orderly flow of medical supplies in the case of public health emergencies.

In addition, the government can raise the cost of coordination arising from the fighting of the hospital by expanding media exposure and taking other measures. It will also help the GNPO make a scientific allocation plan of medical supplies and promote the rational and orderly circulation for medical supplies.

Finally, the accountability of higher authorities for the loose supervision of government is critical to ensuring the rational circulation of medical supplies during public health emergencies. In addition, increasing the cost of strict government supervision and decreasing the cost of loose government supervision are also effective ways to avoid the unscientific allocation of medical supplies during public health emergencies.

During public health emergencies, the rational and orderly circulation of emergency medical supplies is of great importance for the timely treatment of affected people and the rapid control of such events. This not only significantly reduces the economic losses caused by social disorder, such as sick people being unable to work and enterprises temporarily closing down due to the emergency, but also significantly contributes to the subsequent economic recovery.

## Conclusions

Due to the emergency nature of public health emergencies and the enormous devastation they cause, quick emergency measures can prevent greater damage. GNPO, as the hub for the allocation of emergency medical supplies, is essential to ensuring the rapid and scientific circulation of emergency medical supplies. However, due to the lack of emergency medical supplies at this time, it is a common challenge for countries around the world to coordinate the relevant stakeholders. In this paper, a tripartite evolutionary game model of GNPO-hospital-government is constructed. The results of the study suggest that a reasonable reward and punishment mechanism should be established for the stakeholders involved in the circulation of emergency medical supplies. At the same time, GNPO should be encouraged to scientifically allocate medical supplies, hospital should accept GNPO’s allocation plan, and the government should strictly supervise the allocation process. By increasing the cost of conflict, reducing the willingness to conflict, and promoting the cooperation of relevant stakeholders, the scientific and orderly circulation of emergency medical supplies can be effectively ensured.

Specifically, as the allocator of medical supplies, the GNPO should choose a rational allocation strategy that balances efficiency and equity during public health emergencies. It is simpler to achieve the goal of maximizing social benefits by allocating limited emergency supplies to demand points that match the degree of urgency. For example, in COVID-19, emergency medical supplies should be prioritized for allocation to government-designated fever hospitals that have a greater need for medical supplies and greater treatment capacity. This also requires the GNPO to improve its ability to utilize resources and maximize the value of scarce resources.

Hospitals should also actively choose the strategy of not fighting the GNPO’s allocation plan, while strengthening trust in the GNPO and reducing conflict, so that social benefits can be maximized. In addition, in a public health emergency, hospitals may not be able to obtain medical supplies from GNPOs to meet their needs. Considering the cost of fighting and its negative impact on themselves and society, they can also seek other channels to supplement their own needs for medical supplies instead of spending their time on fighting.

The government should strengthen the supervision of the medical supply circulation process during public health emergencies, and it should utilize its credibility to accelerate mutual trust between GNPOs and hospitals so that it can improve rescue efficiency. First, it should ensure hospitals’ own interests while increasing penalties for troublemakers who use irrational comparison psychology to maximize their own interests. Effective incentive mechanisms should also be explored to transform conflicts into symmetrical incentives that can help reduce hospitals’ motivation to fight. Secondly, GNPO should be monitored for timely and effective disclosure of information related to the process of medical supply allocation. Finally, in situations of public health emergencies, when GNPOs cannot guarantee the meeting of hospitals’ medical supply needs, the government can consider increasing the number of medical supply allocators, such as other social organizations, to make the allocation of medical supplies more efficient during public health emergencies.

This study is one of the first to explore the competitive relationship between the GNPO, hospital, and government in the allocation process of emergency medical supplies during public health emergencies. It not only provides a theoretical supplement to the research related to the circulation of emergency resources during public health emergencies, but also provides practical guidance for the specific behavioral strategies of GNPO, hospital, and the government.

However, this paper only considers the influence of the government and hospital on the circulation of medical supplies and the supervision of the circulation process under the condition of incomplete information, and does not consider other influencing factors such as transport time on the circulation of medical supplies during public health emergencies, or the influence of the game order on the game outcome. Therefore, in future research, we can introduce more players and consider relevant influencing factors to study the circulation mechanism of medical supplies in the case of public health emergencies, in order to get more valuable research results.

## Data Availability

All the data included in this study are available upon reasonable request by contacting the corresponding authors.
